# Screening-based approach to discover effective platinum-based chemotherapies for cancers with poor prognosis

**DOI:** 10.1371/journal.pone.0211268

**Published:** 2019-01-29

**Authors:** Hristo P. Varbanov, Fabien Kuttler, Damiano Banfi, Gerardo Turcatti, Paul J. Dyson

**Affiliations:** 1 Institut des Sciences et Ingénierie Chimiques, Ecole Polytechnique Fédérale de Lausanne (EPFL), Lausanne, Switzerland; 2 Biomolecular Screening Facility, Ecole Polytechnique Fédérale de Lausanne (EPFL), Lausanne, Switzerland; Columbia University, UNITED STATES

## Abstract

Drug combinations are extensively used to treat cancer and are often selected according to complementary mechanisms. Here, we describe a cell-based high-throughput screening assay for identification of synergistic combinations between broadly applied platinum-based chemotherapeutics and drugs from a library composed of 1280 chemically and pharmacologically diverse (mostly FDA approved) compounds. The assay was performed on chemoresistant cell lines derived from lung (A549) and pancreatic (PANC-1) carcinoma, where platinum-based combination regimens are currently applied though with limited success. The synergistic combinations identified during the screening were validated by synergy quantification using the combination index method and via high content fluorescent microscopy analysis. New promising synergistic combinations discovered using this approach include compounds currently not used as anticancer drugs, such as cisplatin or carboplatin with hycanthone and cisplatin with spironolactone in pancreatic carcinoma, and carboplatin and deferoxamine in non-small cell lung cancer. Strong synergy between cisplatin or carboplatin and topotecan in PANC-1 cells, compared to A549 cells, suggests that this combination, currently used in lung cancer treatment regimens, could be applied to pancreatic carcinoma as well. Several drugs used to treat diseases other than cancer, including pyrvinium pamoate, auranofin, terfenadine and haloprogin, showed strong cytotoxicity on their own and synergistic interactions with platinum drugs. This study demonstrates that non-obvious drug combinations that would not be selected based on complementary mechanisms can be identified via high-throughput screening.

## Introduction

**Fig 1:**
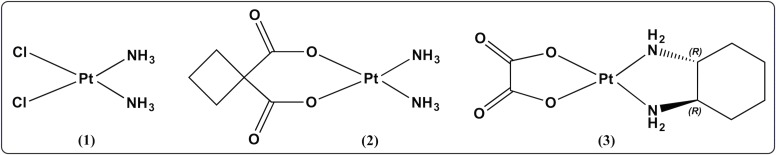
Platinum complexes with worldwide clinical approval: cisplatin (1), carboplatin (2) and oxaliplatin (3).

Cancer, one of the leading causes of death, is a term used to describe a large group of related diseases featuring over 100 subtypes, which differ significantly in terms of incidence, mortality and prevalence [1–4]. Advances in treatment strategies together with earlier diagnosis have systematically increased the survival of cancer patients [5,6]. Amongst these advances, the introduction of cisplatin has arguably led to one of the key improvements. For example, the cure rate of patients diagnosed with testicular cancer increased from 10 to over 95% after the introduction of cisplatin (FDA approval in 1978) [7–9]. At present, cisplatin and its analogues carboplatin and oxaliplatin (Fig 1) are extensively used in cancer chemotherapy, usually in combinations with other drugs [8,10–12]. Indeed, 36 of 75 anticancer treatment regimens listed in Martindale drug reference book are platinum-based [13], while Pt drugs are prescribed to nearly halve of the patients undergoing chemotherapy in Australia [12]. Furthermore, preclinical and clinical efficacy of new anticancer drug candidates is commonly trialed in combination with platinum chemotherapeutics [11,14].

The clinically used platinum-based drugs are neutral Pt(II) complexes with a *cis* square planar geometry bearing so-called carrier ligands (i.e. two ammines or chelating diamine) and leaving groups (two chlorides or chelating dicarboxylate) [7,14–16]. Their mechanism of action includes intracellular activation by aquation and subsequent covalent binding to DNA bases, which primarily leads to cell death by apoptosis [7,15]. The main limitations to their use include severe side effects and resistance (intrinsic in some tumors or acquired during chemotherapy) [11,15,17].

Cisplatin-resistant cancer types usually have poor prognosis and a high mortality. Pancreatic adenocarcinoma, for example, has a 5-years survival rate for patients of only 2.4% [4,18,19]. Surgical resection offers the only potentially curative treatment, but in the majority of the cases the disease is inoperable and metastatic at the time of diagnosis. Therefore, chemotherapy (i.e. gemcitabine-based combinations) remains the standard-of-care in most countries, as it improves the overall survival and quality of life of patients [19]. Pancreatic cancer does not respond well to Pt drugs, although cisplatin and oxaliplatin are sometimes used in the combination therapy [19–21].

Lung carcinoma is the leading cause of cancer death worldwide [4,22], despite the number of treatment regimens available [13,21]. Pt drugs are amongst the most useful agents used to treat lung cancer and first-line chemotherapy typically involves cisplatin or carboplatin in combination with other antineoplastic drugs (e.g. paclitaxel, gemcitabine, docetaxel, vinorelbine, irinotecan or pemetrexed) [20,21,23]. Nevertheless, gains in overall survival are marginal (5-year survival of 10%) [18] and development of more effective platinum-based combinations is necessary. Recently, targeted therapies have also been introduced for lung cancer treatment. Epidermal growth factor receptor (EGFR) inhibitors have been successfully used as co-drugs to improve the prognosis of non-small cell lung cancer (NSCLC) expressing EGFRs. Although the subset of NSCLCs responding to this treatment is small, the results demonstrate that improvements can be made by tailoring therapy in a cancer type specific manner [24,25].

We have recently shown that new potential chemotherapeutics for the treatment of problematic malignancies such as lung and pancreatic cancers can be identified by screening libraries containing drugs already approved for other applications [26]. A key advantage of such an approach (often referred to as drug repurposing or repositioning) [27] is that all recognized hits are compounds with confirmed safety and bioavailability in humans and subsequently clinical development costs are lower, with shorter approval times and higher approval rates [27,28]. We have further expanded our methodology with the aim of identifying drug-like molecules that can synergistically potentiate the cytotoxic effects of clinically applied Pt drugs (see Fig 1) against cancers with poor prognosis. This is of particular utility, as anticancer chemotherapies typically employ drug cocktails, rather than a single agent. The selection of anticancer agents for combination therapy is usually done based on the complementary mechanisms of the drugs and non-overlapping toxic side-effects, while sometimes established regimens are adapted from one cancer type to another [29,30].

In this study, we present an alternative, screening-based approach that identifies promising and previously unidentified anticancer drug combinations for platinum-based treatments of specific cancer types. High-throughput screening (HTS) assay was applied to chemoresistant A549 (NSCLC) and PANC-1 (pancreatic carcinoma) cell lines and has demonstrated that novel combination therapies may be able to overcome resistance mechanisms to Pt drugs. Potential synergistic combinations identified during the screening were confirmed and further validated, e.g. synergy quantification using the combination index (CI) method of Chou and Talalay [31] and High Content Analysis (HCA) using automated fluorescent microscopy. Selected combinations were further examined in HCT116 (colorectal carcinoma) cells, as well as in healthy counterparts of carcinoma models, such as MRC-5 (normal lung fibroblasts) and BJ (normal foreskin fibroblasts) cell lines. The data suggests that for certain drug combinations, doses could be lowered without compromising efficacy, potentially reducing the side effects. Although extrapolation from cell culture to in vivo models often requires further optimization, in vitro drug combination studies could provide valuable information in the development of new treatment regimens [32]. The methodology presented herein has considerable potential for optimizing Pt drug anticancer therapy including the possibility to obtain new combination treatments for chemoresistant cancers.

## Results and discussion

**Fig 2:**
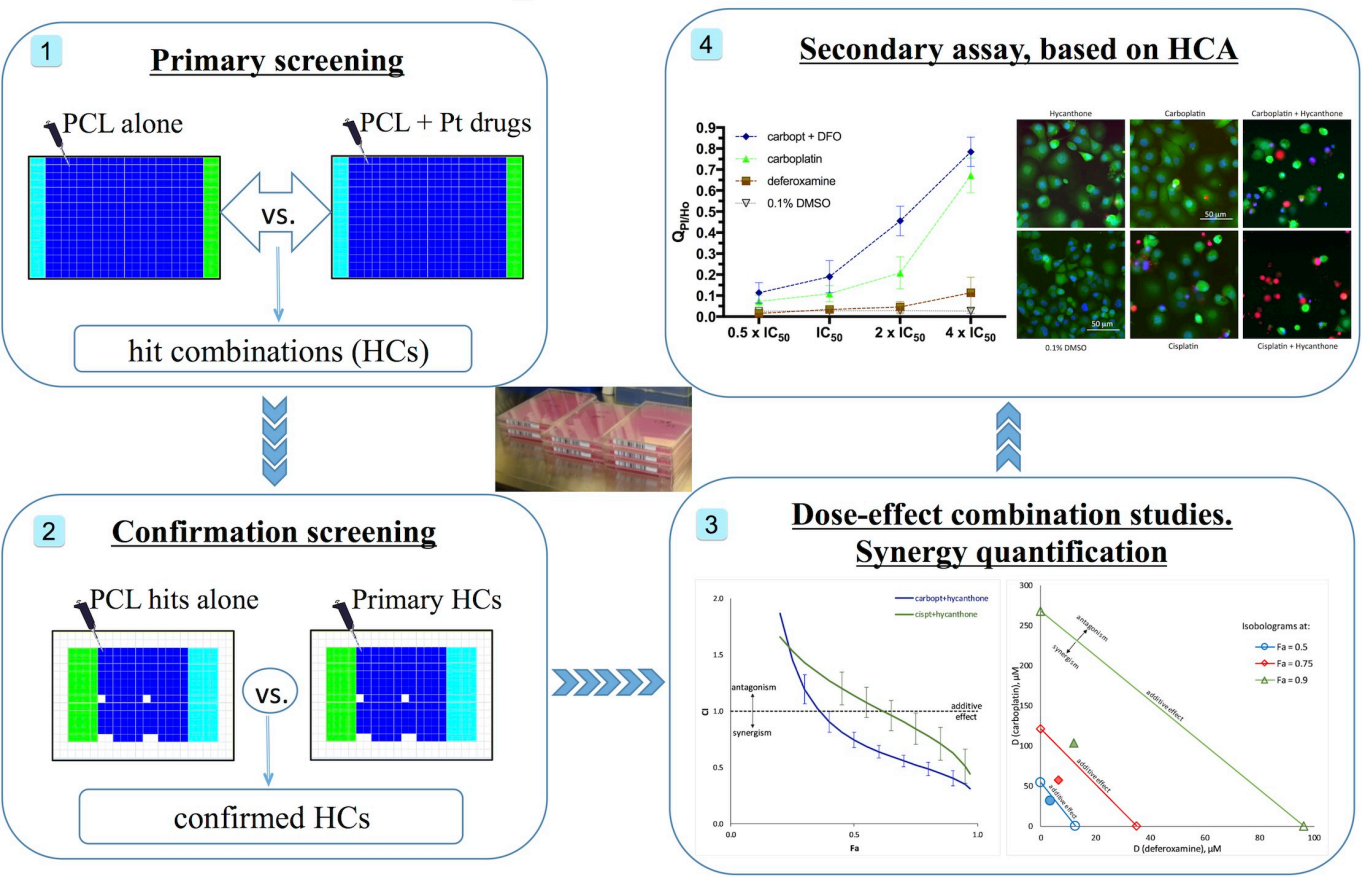
**Schematic overview of the workflow used in this study**: 1) Screening of Prestwick Chemical Library (PCL) alone and in combination with one of the approved Pt drugs against PANC-1 and A549 cells to identify hit combinations (HCs); 2) Re-testing the PCL compounds identified in HCs during the primary study alone and in combination with the Pt drug to exclude false-positive HCs; 3) HCs studies in dose-response manner and synergy quantification; 4) Secondary assay, based on HCA.

Combinations of Pt drugs (see Fig 1) and other known drugs, which act synergistically against PANC-1 and A549 cells were identified in this study following a step-wise protocol (summarized in Fig 2). The method can easily be applied to other cancer cell types that grow in culture.

### Primary HT screening–identification of HCs

The primary screening step comprises a commercial library of approved drugs to be screened in combination with Pt drugs for activity against specific cancer cell lines. The PCL [33], which is composed of 1280 chemically and pharmacologically diverse compounds (ca. 90% being FDA-approved drugs) was selected. Experimental conditions (i.e., number of cells per well, concentration of the library compounds, drugs exposure time, volumes of the reagents used) for the screening assays were adapted from an established and validated protocol for screening of PCL against PANC-1 and A549 cells [26]. The PCL compounds (at a 10 μM single point concentration) are screened alone and in combination with cisplatin, carboplatin or oxaliplatin (Fig 1), at concentrations chosen to correspond to their IC_50_ and IC_20_ values (as determined in the preliminary assays, see S1 Fig). The assay protocols were optimized in order to minimize any detrimental effects of DMSO (from the PCL stock solutions) on the activity of the Pt drugs [34,35]. Thus, PCL compounds were dispensed first into empty plates, followed by addition of the cell suspension and then the Pt drug last (freshly prepared in water or PBS stocks, diluted with medium). The final concentration of DMSO was therefore 0.1% and its direct interaction with the Pt drugs was limited by the high dilution in medium (see also S2 Fig). The screening experimental protocol is summarized in Table 1 and depicted in S3 Fig.

**Table 1:** Summary of the parameters/conditions used in the primary HTS assay.

Cell line	PANC-1	A549
Cell suspension volume	25 μl/well in medium	
Cell number	2000 cells/well	
PCL compounds/controls	30 nl in DMSO	
C_tot_ (PCL compounds)	10 μM, 0.1% DMSO	
V (Pt drugs/PBS/water)	5 μl/well in medium	
C_tot_ (cisplatin) Ctrl/IC_20_/IC_50_	0/5/15 μM	0/1.5/7 μM
C_tot_ (carboplatin) Ctrl/IC_20_/IC_50_	0/20/100 μM	0/15/90 μM
C_tot_ (oxaliplatin) Ctrl/IC_20_/IC_50_	0/2/16 μM	0/0.5/2.5 μM
Drugs exposure time	72 h	50 h
V (PrestoBlue)	3 μl/well, 1 h incubation time	

The results from every screen plate were normalized to the controls obtaining HTS scores, where a score of 0 corresponds to the average fluorescence intensity of the negative control wells (cells + 0.1% DMSO) and a score of 1 to that of the positive control wells (cells + 10 μM Doxorubicin) for each plate. In the case of the PCL-Pt combination plates, a fixed concentration of the respective Pt drug is added to every well (including those with positive and negative controls). Subsequently, the calculation of the scores is based on normalization to the effect of the Pt drug alone (at its IC_20_ or IC_50_ value). This approach allows potential synergistic combinations between the Pt drugs and the PCL compounds to be identified from the direct comparison between the scores of the PCL compounds alone and their combinations with the respective Pt drugs. A higher score of a PCL compound in combination with certain Pt drug, compared to the score of the PCL compound alone suggests more than cumulative cytotoxicity (i.e. synergistic), and such drug combinations are marked as ‘hit-combinations’ (HCs). Over 25 HCs for PANC-1 cells and 50 HCs in A549 cells were identified during the primary screen (S4 Fig). In a few cases, an effect on cell viability was noticed only in the presence of a Pt drug (HTS scores, higher than the average of the negative controls + 3 x SD were detected only in the PCL-Pt combination plates). All HCs found during the primary screen were subjected to a confirmation screen (to exclude false-positive HCs), employing the same experimental protocol used in the primary screen. In the cases where synergistic interactions could not be revealed due to the high activity of the library drug on its own (primary scores > 0.9; e.g. anthracyclines, cardiac glycosides, disinfectants), the experimental setting was modified and the respective PCL compounds were tested at 12 times lower concentration (0.83 μM instead of 10 μM).

### Confirmation screening

About 60% of the HCs identified during the primary screening step were confirmed in the confirmation screening (see Table 2 for list of the confirmed HCs). In addition, the lower concentration setting enabled the identification of HCs with very active PCL compounds, i.e. daunorubicin and camptothecine. Many of the false-positive primary HCs, which were not confirmed during the confirmation screen, could be explained with the very steep concentration-effect relationships and/or large variability of the response for some PCL compounds, e.g. thiostreptone, verteporfin, haloprogin, tegaserod (see also ref. [26]). It should also be noted that not all synergistic combinations identified for cisplatin could be found for carboplatin (and vice versa), despite both drugs having the same spectrum of anticancer activity. This finding can be attributed to the different rates and mechanisms of activation of cisplatin and carboplatin, reflected in different cellular pharmacokinetics.

**Table 2:** Confirmed HCs.

cells	cisplatin	carboplatin	oxaliplatin
**PANC-1**	Beta-Escin^c^, Camptothecine (S,+)^d^, Daunorubicin^a^^,^^c^, Hycanthone^c^, Pyrvinium pamoate^c^, Spironolactone^c^, Topotecan^d^, Vorinostat^c^	Aminacrine^c^, Camptothecine (S,+)^c^, Daunorubicin^a^^,^^c^, Hycanthone^c^, Pyrvinium pamoate^c^, Topotecan^c^	Pyrvinium pamoate^c^, Suloctidil^c^, Topotecan^c^,
**A549**	Auranofin^c^, Azacytidine-5^c^, Camptothecine (S,+)^a^^,^^d^, Cladribine^c^, Cytarabine^c^, Gemcitabine^c^, Pyrvinium pamoate^d^, Topotecan^d^, Vorinostat^d^,	Aminacrine^d^, Auranofin^c^, Azacytidine-5^b^, Cytarabine^c^, Daunorubicin^a^^,^^b^, Deferoxamine^b^ Floxuridine^b^, Flumethasone^b^, Gemcitabine^d^, Pyrvinium pamoate^c^	Auranofin^d^, Camptothecine (S,+)^a^^,^^d^, Erlotinib^b^, Fluvastatin^b^, Gemcitabine^b^, Haloprogin^c^, Terfenadine^d^, Topotecan^d^, corticosteroids^b^^,^^e^

^a^ identified only in the c(PCL) = 0.83 μM experimental setting

^b^ HCs with the Pt drug at c ≈ IC_20_ μM

^c^ HCs with the Pt drug at c ≈ IC_50_ μM

^d^ HCs with the Pt drug at both concentrations

^e^ corticosteroid drugs: triamcinolone, prednicarbate, hydrocortisone base, prednisolone, flumethasone, fluocinolone acetonide, deflazacort, flunisolide, fludrocortisone acetate, dexamethasone acetate and desonide.

#### Combinations with known anticancer drugs

Cytotoxic antibiotics, such as the positive control doxorubicin, are the most cytotoxic anticancer agents included in the library [26]. They caused significant to complete inhibition of cell viability (scores > 0.9*)* in both cell lines at the primary screening concentration setting. Re-testing them at 12-times lower concentration allowed the identification of HCs between cisplatin/carboplatin and daunorubicin in both cell lines (S5 Fig). Anthracyclines, such as daunorubicin and doxorubicin, are used and have been trialed in a number of combination chemotherapy regimens against a wide range of malignancies including lung cancer [13,21,22,36,37]. Moreover, cisplatin and doxorubicin in combination are part of treatment regimens for ovarian, stomach and bladder cancers [13,22]. Our results support that combination therapies featuring Pt drugs and anthracyclines may have potential to improve existing treatment options for pancreatic and lung cancers.

Several HCs involving antimetabolites, e.g. azacytidine-5, cladribine, cytarabine and gemcitabine, and one or more of the Pt drugs were detected in A549 cells. Most of these HCs were validated during the confirmation screen (see S6 Fig) and the following dose-response studies (see below). Antimetabolites are S-phase specific chemotherapeutic agents, frequently employed together with Pt drugs in the treatment regimens of various cancers [13,22]. Indeed, chemotherapy schedules for lung and pancreatic cancers also include combinations of Pt drugs and antimetabolites, particularly gemcitabine [21]. However, no HCs including antimetabolites were detected in PANC-1 cells during the screens, emphasizing the need for new therapeutic approaches to treat pancreatic cancer.

**Fig 3:**
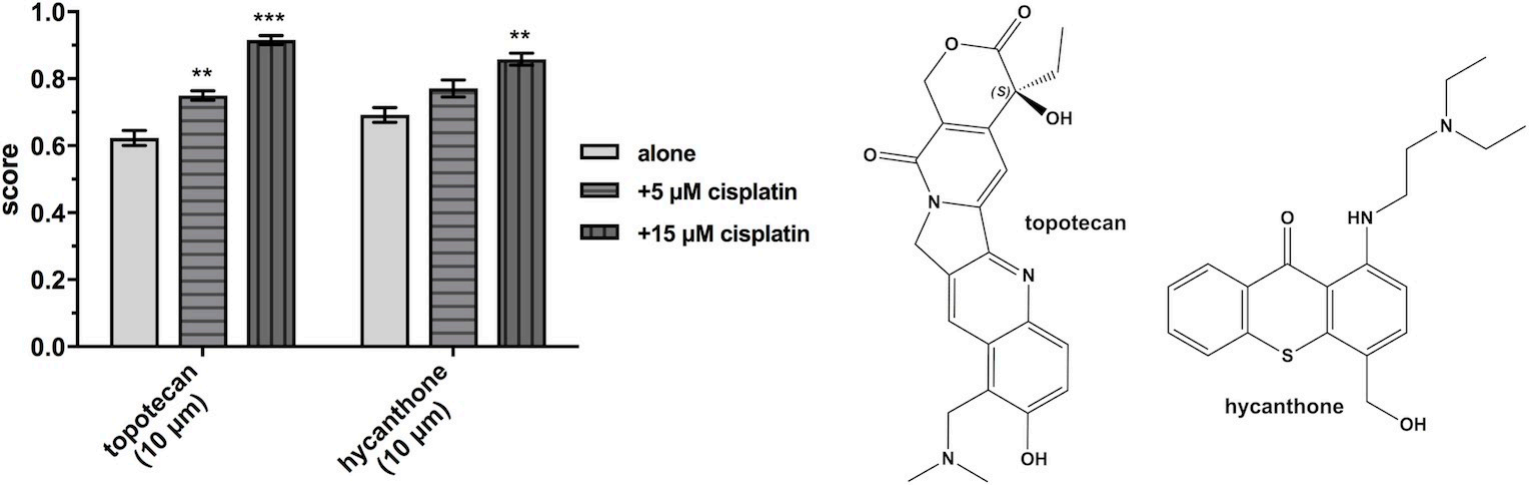
HTS scores of topotecan and hycanthone alone and in combinations with cisplatin, obtained from the confirmation screen in PANC-1 cells. Data is presented as means ± SDs from 2 replicates. Statistical significance is determined by ordinary one-way ANOVA followed by Dunnett’s multiple comparison test (**p < 0.01 and ***p < 0.001) using GraphPad Prism 7. Chemical structures of topotecan and hycanthone (right).

The topoisomerase I inhibitors camptothecine and topotecan synergize with the platinum drugs in both PANC-1 and A549 cells. Higher cytotoxicity of cisplatin in combinations with camptothecines and anthracyclines in lung cancer cells was demonstrated also by recent studies [38,39]. Indeed, topotecan is used in combination with cisplatin or carboplatin in chemotherapeutic regimens for lung (and other) cancers [13,21]. Thus, the results from the screens confirm the synergistic basis for the use of gemcitabine-platinum and topotecan-platinum regimens for treatment of lung cancer and reflect the applicability of the screening approach and A549 cells as a model. Furthermore, the data obtained in PANC-1 cells (see Fig 3 and “synergy quantification”, Table 3) suggest that topotecan-platinum treatments could be expanded to pancreatic cancer.

Other confirmed HCs include the protein kinase inhibitor erlotinib [40] and oxaliplatin in A549 cells, and the histone deacetylase inhibitor (HDACi) vorinostat and cisplatin in both cell lines. Vorinostat is used for the treatment of cutaneous T cell lymphoma and is currently undergoing early-phase clinical trials for lung and pancreatic cancers [41–43]. Moreover, recent studies found an enhanced antitumor effect for the combination of vorinostat with cisplatin in lung cancer models [38,44,45].

Surprisingly, the plant alkaloids paclitaxel and docetaxel, which are commonly employed in platinum-based treatment regimens [13], showed decreased scores in the presence of the Pt drugs (less than additive effect) in the screens. A similar effect was observed also for other mitotic inhibitors not currently used in cancer therapy, i.e. colchicine and podophyllotoxin. The lower scores obtained for combinations between taxanes and Pt drugs might be linked to the reverse mode of the screening assay, as the mode of action of mitotic inhibitors may vary for attached cells and for cell suspensions and in some cases even from the concentration used. Furthermore, taxanes are usually applied at much lower concentrations than these used in the assay (IC_50_ values are commonly in the nanomolar range [26,31]).

#### Combinations with non-anticancer drugs

The discovery of HCs with drugs used to treat diseases other than cancer is of interest due to the possibility of repositioning existing non-cytotoxic drugs in novel combinations with Pt drugs. Such combinations can be translated to cancer therapy relatively rapidly and at lower cost than introducing new anticancer drugs [27].

Cardiac glycosides and their aglycones represent the most active class of non-anticancer drugs in the PCL, exhibiting strong cytotoxic effects in both cell lines that are retained at the lower concentration setting of the confirmation screen. The potential of cardiac glycosides as anticancer drugs, and their main limitations associated with their narrow therapeutic indices and low maximum tolerated dose (MTD)s, have been reported [46–49]. In this context, synergistic combinations with other drugs that can decrease the required doses of cardiac glycosides are needed to access their antitumor effects for clinical relevance. Indeed, Felth et al. reported additive to slightly synergistic interactions between different cardiac glycosides and 5-FU, cisplatin or oxaliplatin in colon cancer cells [49]. Nevertheless, HCs between the Pt drugs and cardiac glycosides could not be identified under the high-throughput conditions employed in our study due to the strong cytotoxic effects of cardiac glycosides on their own, even at the low concentration setting. Further dose-effect combination studies with the cardiac glycosides aglycones digoxigenin and digitoxigenin and the Pt drugs revealed additive to antagonistic interactions in PANC-1 and A549 cells; however, the steep dose-response curves of digoxigenin and digitoxigenin impeded accurate synergy quantification.

The anthelmintic, pyrvinium pamoate, appeared in HCs with all Pt drugs in both cell lines in the primary and confirmation screens. Unfortunately, further dose-response studies for synergy quantification were impeded by solubility problems and precipitation of the drug after cell seeding. Similar solubility-related issues were observed with haloprogin and terfenadine. Another antiparasitic drug, the schistosomicide hycanthone, showed enhanced cytotoxicity in PANC-1 cells in combination with cisplatin (Fig 3) and carboplatin and these combinations were subjected to further dose-response studies (see next paragraphs). Interestingly, no HCs featuring the collection of benzimidazole athelmintics (some of which are currently being repositioned as anticancer drugs) [26,50,51] were found during the screens. Moreover, in most of these cases (e.g., parbendazole, albendazole, mebendazole), the anthelmintic drugs displayed lower scores in the presence of Pt drugs.

The antirheumatic agent, auranofin, synergizes with all three Pt drugs in A549 cells. Auranofin is a gold complex, which potential as anticancer drug was demonstrated in several preclinical studies and it is currently examined in clinical trials for treatment of leukemia and some advanced solid tumors [52–54], and has also been show to synergize with ruthenium anticancer drug candidates [55].

Other PCL compounds identified and confirmed in HCs with one or more of the Pt drugs and advanced to further validation studies include the acridine derivative, aminacrine, the saponine mixture, beta-escin, the iron chelator, deferoxamine, the anti-hyperlipidemic drug, fluvastatin and the peripheral vasodilatator, suloctidil. (see Table 2)

Several corticosteroid drugs showed higher scores in the presence of oxaliplatin and/or carboplatin in A549 cells, while in some cases cytotoxicity was observed only in the PCL-Pt combination plates. Most of the steroid drugs found in the confirmed HCs showed only marginal and dose-independent cytotoxicity in A549 cells when tested alone [26]. The ability to potentiate the cytotoxicity of Pt drugs towards A549 cells was further evaluated for some of them (see below). Corticosteroids are included in several cancer chemotherapy regimens [56], and combination of a Pt(IV) complex, satraplatin, and the corticosteroid, prednisone, was investigated in clinical trials for the treatment of prostate cancer [57].

The potassium-sparing diuretic with steroid structure, spironolactone (S7 Fig), enhances the activity of cisplatin against PANC-1 cells, without displaying activity on its own under the screening conditions. Our findings are in agreement with recent reports on the ability of spironolactone to sensitize ovarian and colon cancer cells by inhibition of different DNA repair mechanisms [58,59]. Spironolactone shows no cytotoxic activity in a large panel of cancer cell lines [60], however, it exhibits some anti-angiogenic properties [61]. Its effects on the activity of cisplatin in PANC-1 cells were further evaluated.

### Dose-effect combination studies (synergy quantification)

**Fig 4:**
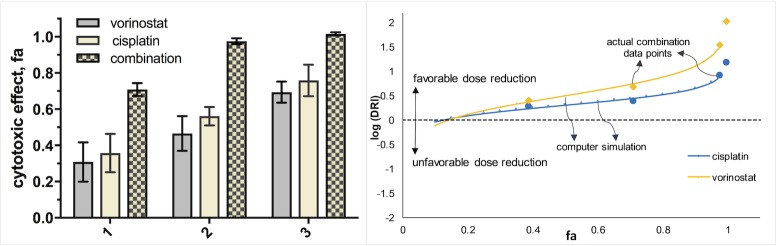
Cytotoxic effects of vorinostat and cisplatin in PANC-1 cells, alone and in combination at concentrations: 1 (7.5 and 10 μM), 2 (15 and 20 μM) and 3 (30 and 40 μM) after 72 h of exposure. Data is presented as means ± SDs from 4 replicates (vorinostat and combination) or 8 replicates (cisplatin) per concentration. Dose-reduction index (DRI) plot is shown on the right.

**Fig 5:**
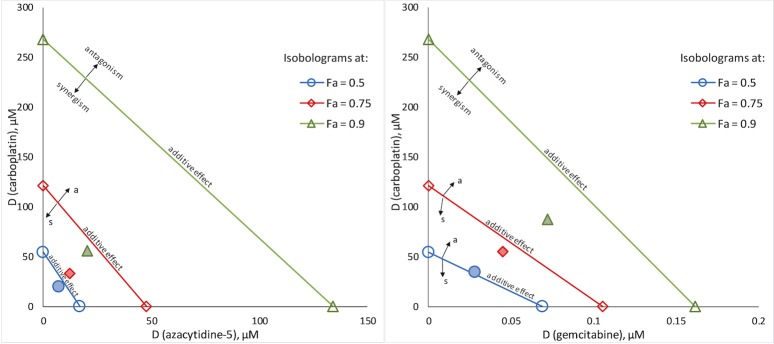
Synergistic combinations between carboplatin and antimetabolites in A549 cells. Classical isobolograms at 0.5, 0.7 and 0.9 effect level (IC_50_, IC_75_ and IC_90_ concentrations, respectively) for the combinations of carboplatin with azacytidine-5, 1:0.37 (left), and with gemcitabine, 1:0.001 (right); markers for the actual combination points are pattern filled.

**Fig 6:**
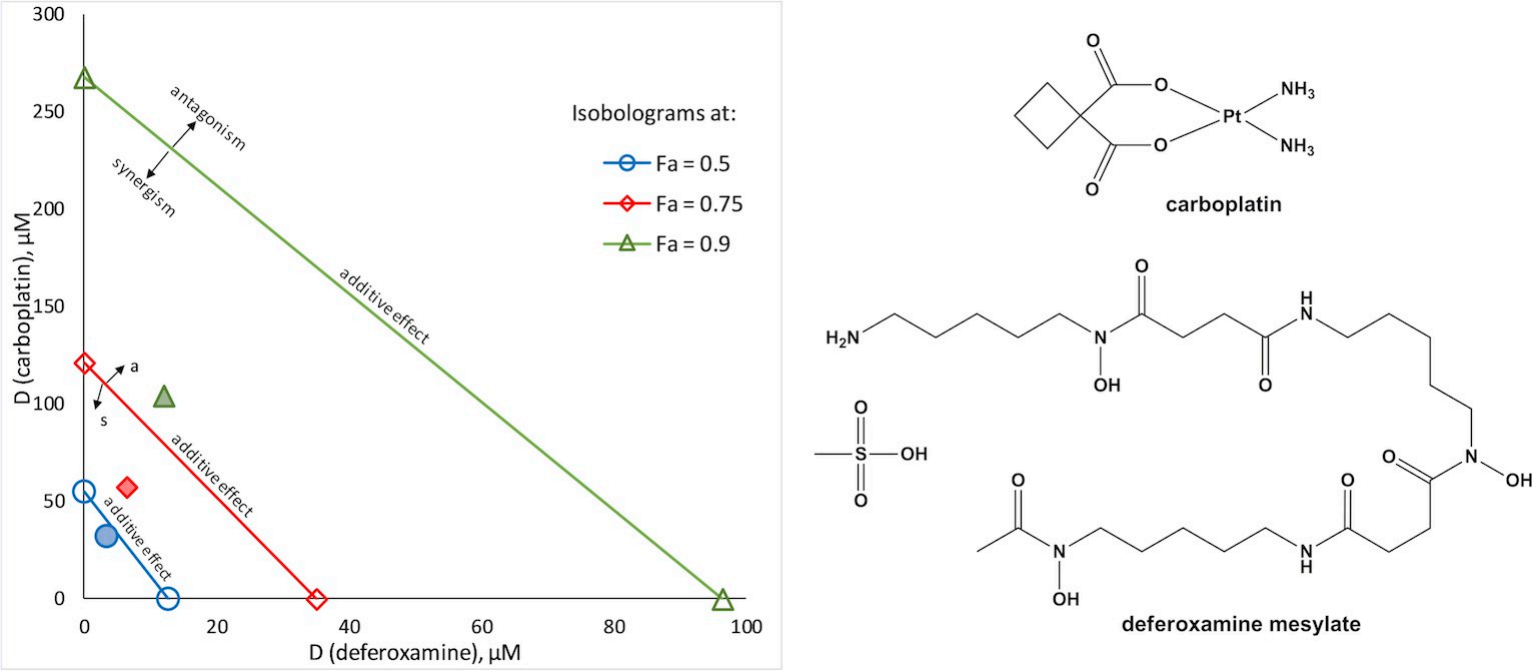
Synergistic combination of carboplatin and deferoxamine (1:0.12) in A549 cells. Classical isobologram at 0.5, 0.7 and 0.9 effect level (IC_50_, IC_75_ and IC_90_ concentrations, respectively); markers for the actual combination points are pattern filled; chemical structures are shown on the right.

Quantification of synergism for the confirmed HCs was assessed using the median-effect principle—combination index (MEP-CI) method with a diagonal constant ratio experimental design [31,32,62]. The results from the combination analysis are summarized in Tables 3 and 4 and selected diagrams and isobolograms are shown in Figs 4–6 and in the ESI (S8–S10 Figs). As synergism and antagonism usually differ at different dose/effect levels, the CI values for the tested combinations are presented at high effect levels, which are in principle of higher therapeutic relevance for cancer chemotherapy [31].

**Table 3:** CI values for the investigated combinations in PANC-1 cells.

Compound combination	Combination ratio	CI values^a^ at effect level of		CI_wt_ values^b^	Assigned symbol^c^
		75%	90%		
cisplatin + beta-escin	1 : 1.247	1.007 (1.015 +/- 0.239)	0.826 (0.845 +/- 0.320)	0.868	+
cisplatin + camptothecine	1 : 0.083	0.345 (0.341 +/- 0.069)	0.245 (0.242 +/- 0.071)	0.376	+++
cisplatin + daunorubicin	1 : 0.022	0.680 (0.670 +/- 0.147)	0.449 (0.453 +/- 0.149)	0.552	+++
cisplatin + hycanthone	1 : 0.250	0.837 (0.836 +/- 0.139)	0.614 (0.616 +/- 0.150)	0.665	+++
cisplatin + topotecan	1 : 0.250	0.395 (0.392 +/- 0.070)	0.277 (0.275 +/- 0.075)	0.340	+++
cisplatin + vorinostat	1 : 0.749	0.495 (0.492 +/- 0.074)	0.305 (0.303 +/- 0.068)	0.361	+++
carboplatin + aminacrine	1 : 0.034	0.380 (0.389 +/- 0.097)	0.265 (0.276 +/- 0.109)	0.302	+++
carboplatin + daunorubicin	1 : 0.010	0.974 (0.976 +/- 0.182)	0.630 (0.638 +/- 0.220)	0.727	++
carboplatin + hycanthone	1 : 0.101	0.524 (0.528 +/- 0.054)	0.408 (0.414 +/- 0.068)	0.351	+++
carboplatin + topotecan	1 : 0.084	0.095 (0.097 +/- 0.023)	0.018 (0.019 +/- 0.008)	0.077	+++++
oxaliplatin + topotecan	1 : 1.265	0.819 (0.836 +/- 0.241)	0.824 (0.899 +/- 0.769)	0.827	++

^a^ CI values were calculated using CompuSyn [62]; the values in brackets represent the means ± 95% confidence intervals, as determined from the sequential deletion analysis (S.D.A.) as a measure of the CI variability at the presented effect levels (i.e., at IC_75_ and IC_90_).

^b^ Weighted average CI values, calculated as follow: CI_wt_ = (CI_50_ + 2CI_75_ + 3CI_90_ + 4CI_95_)/10.

^c^ Degrees of synergism (+ signs) or antagonism (- signs) are based on the ranges of CI_wt_ values as described in ref. [31]; (±) near additive effect, (+) slight synergism, (++) moderate synergism, (+++) synergism, (++++) strong synergism and (+++++) very strong synergism; antagonism is divided in the same way, except using “-”signs.

**Table 4:** CI values for the investigated combinations in A549 cells.

Compound combination	Combination ratio	CI values^a^ at effect level of		CI_wt_ values^b^	Assigned symbol^c^
		75%	90%		
cisplatin + auranofin	1 : 1.555	1.133 (1.129 +/- 0.191)	1.008 (1.005 +/- 0.148)	1.061	±
cisplatin + azacytidine-5	1 : 3.721	0.484 (0.486 +/- 0.125)	0.262 (0.273 +/- 0.145)	0.337	+++
cisplatin + camptothecine	1 : 0.033	0.701 (0.699 +/- 0.057)	0.460 (0.455 +/- 0.052)	0.523	+++
cisplatin + cladribine	1 : 0.191	0.693 (0.694 +/- 0.178)	0.378 (0.385 +/- 0.171)	0.490	+++
cisplatin + gemcitabine	1 : 0.009	0.811 (0.818 +/- 0.183)	0.500 (0.516 +/- 0.230)	0.588	+++
cisplatin + vorinostat	1 : 0.688	0.664 (0.664 +/- 0.047)	0.459 (0.459 +/- 0.045)	0.517	+++
cisplatin + topotecan	1 : 0.111	1.093 (1.098 +/- 0.093)	0.990 (0.998 +/- 0.198)	1.008	±
carboplatin + auranofin	1 : 0.135	1.057 (1.114 +/- 0.669)	1.007 (1.123 +/- 1.186)	1.033	±
carboplatin + azacydine-5	1 : 0.370	0.531 (0.538 +/- 0.118)	0.359 (0.373 +/- 0.176)	0.404	+++
carboplatin + cytarabine	1 : 0.008	0.769 (0.769 +/- 0.128)	0.662 (0.666 +/- 0.187)	0.694	+++
carboplatin + gemcitabine	1 : 0.001	0.838 (0.837 +/- 0.166)	0.772 (0.772 +/- 0.210)	0.791	++
carboplatin + daunorubicin	1 : 0.004	0.839 (0.873 +/- 0.492)	0.684 (0.780 +/- 1.006)	0.774	++
carboplatin + deferoxamine	1 : 0.162	0.648 (0.653 +/- 0.173)	0.533 (0.546 +/- 0.232)	0.559	+++
oxaliplatin + auranofin	1 : 4.571	1.154 (1.174 +/- 0.533)	1.045 (1.089 +/- 0.740)	1.076	±
oxaliplatin + camptothecine	1 : 0.107	0.983 (0.983 +/- 0.123)	0.832 (0.838 +/- 0.202)	0.895	+
oxaliplatin + erlotinib	1 : 4.000	1.194 (1.196 +/- 0.194)	1.133 (1.139 +/- 0.332)	1.160	-
oxaliplatin + fluvastatin	1 : 8.600	1.083 (1.081 +/- 0.168)	0.904 (0.909 +/- 0.163)	0.944	±
oxaliplatin + gemcitabine	1 : 0.012	1.482 (1.480 +/- 0.260)	1.320 (1.349 +/- 0.470)	1.349	—
oxaliplatin + topotecan	1 : 0.210	1.111 (1.111 +/- 0.078)	1.339 (1.338 +/- 0.199)	1.336	—

^a^ CI values were calculated using CompuSyn [62]; the values in brackets represent the means ± 95% confidence intervals, as determined from the S.D.A. as a measure of the CI variability at the presented effect levels (i.e., at IC_75_ and IC_90_).

^b^ Weighted average CI values, calculated as follow: CI_wt_ = (CI_50_ + 2CI_75_ + 3CI_90_ + 4CI_95_)/10.

^c^ Degrees of synergism (+ signs) or antagonism (- signs) are based on the ranges of CI_wt_ values as described in ref. [31]; (±) near additive effect, (+) slight synergism, (++) moderate synergism, (+++) synergism, (++++) strong synergism and (+++++) very strong synergism; antagonism is divided in the same way, except using “-”signs.

Dose-effect combination studies confirmed synergism for most of the combinations tested. Prominent synergistic interactions between camptothecines, namely camptothecine and topotecan, and Pt drugs (especially cisplatin and carboplatin) were determined in PANC-1 cells (S8 Fig). However, these effects were much less pronounced in A549 cells, and the combination between oxaliplatin and topotecan even appears to be slightly antagonistic. Other promising combinations in terms of synergism in PANC-1 cells include cisplatin+vorinostat, carboplatin+aminacrine and carboplatin+hycanthone (Fig 4, S8 and S9 Figs). The combination of vorinostat and cisplatin at their IC_50_ values caused 98% inhibition of cell viability. This value corresponds to an 8-fold reduction of the dose of cisplatin and 34-fold reduction of that of vorinostat compared with the doses of each drug alone needed to cause such effect. Vorinostat and cisplatin showed strong synergistic interactions also in A549 cells (Table 4). These findings demonstrate that the cisplatin-vorinostat combination could show promise in the treatment of pancreatic and lung cancers.

The highest degree of synergism determined in A549 cells was for the combinations between antimetabolites, namely azacytidine-5, cladribine, cytarabine and gemcitabine, with cisplatin or carboplatin. Interestingly, gemcitabine was found to act antagonistically with oxaliplatin in A549 cells. Combination regimens including gemcitabine and cisplatin or carboplatin are commonly used in the chemotherapy of NSCLC. CI analysis revealed that certain Pt drug-antimetabolite combinations exhibit stronger synergism than the clinically applied regimens (Table 4, Fig 5 and S10 Fig), and could provide alternatives to the current treatment options. Another combination that displays promising synergistic effects in A549 cells consist of carboplatin and the iron chelator deferoxamine (Fig 6).

Daunorubicin was shown to act synergistically with cisplatin (PANC-1 cells) and carboplatin (in both cell lines). However, sequential deletion analysis (S.D.A.) showed high variability of the CI values, particularly in the A549 cells, and, therefore, the determined degrees of synergism must be taken with caution. Similarly, synergistic interactions between some compounds, for example the gold drug auranofin or beta-escin and the Pt drugs, could not be confirmed unambiguously due to large variations of the cellular response and the steep dose-response curves. In the case of combinations featuring pyrvinum pamoate, terfenadine, suloctidil or haloprogin, synergy quantification was not possible due to high deviations between the replicates and/or low conformity of the median effect plots (r < 0.85). As already mentioned, in some cases these problems could be attributed to the low water solubility of the PCL compound and precipitation after dilution with cell medium.

**Fig 7:**
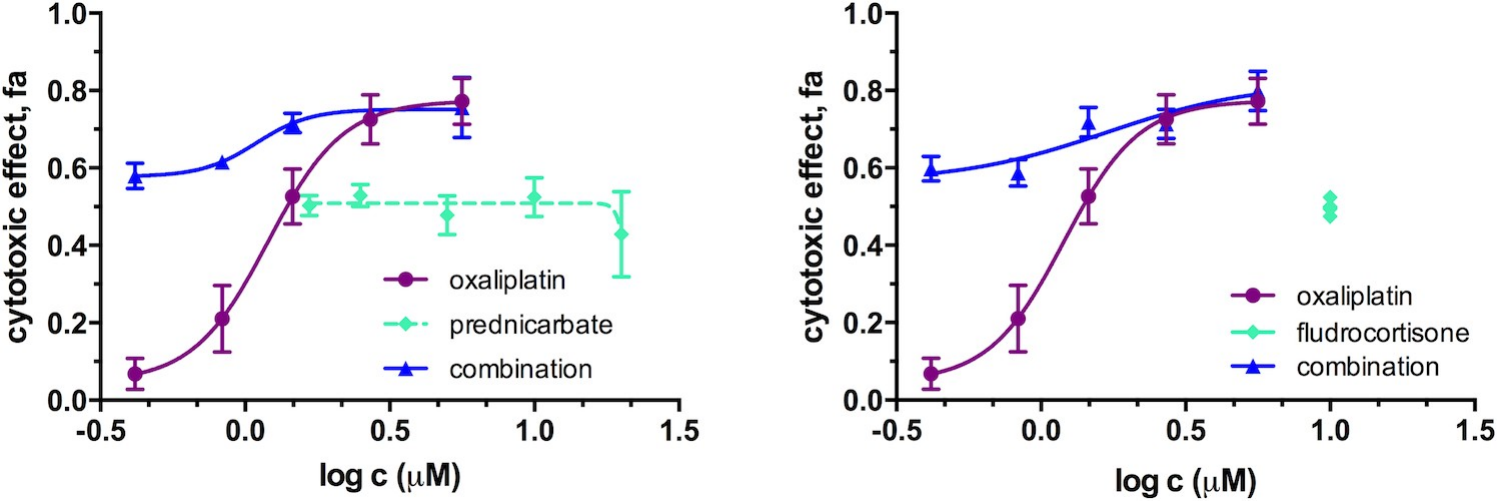
Concentration effect curves of oxaliplatin, alone and in combination with corticosteroids: prednicarbate (at concentration range 1.5–20 μM) and fludrocortisone (at fixed concentration of 10 μM) in A549 cells after 72 h of exposure. Curves fitting and graphs are prepared with GraphPad Prism 7.

The combination effects between Pt drugs (i.e., carboplatin and oxaliplatin) and corticosteroids in A549 cells could not be evaluated with the CI method of Chou-Talalay as the flat dose-response curves (fa ≈ 0.3–0.5 in the range 0.1–100 μM) of the steroids did not allow determination of the parameters required for the analysis (D_m_ and m). However, the fractional product method of Webb [63] revealed no synergistic interactions between the Pt drugs and the steroid drugs at the concentrations tested (see Fig 7, S11 and S12 Figs). As a proof of principle, the same methodology was applied also to evaluate the synergistic combinations cisplatin+vorinostat and carboplatin+topotecan in PANC-1 cells as well as the cisplatin+palcitaxel combination in A549 cells. The fractional product method confirmed the synergy for cisplatin+vorinostat and carboplatin+topotecan combinations and suggested antagonistic interactions between cisplatin and paclitaxel in A549 cells at most of the concentrations tested (S12 Fig) in good agreement with the results obtained from the screens.

**Fig 8:**
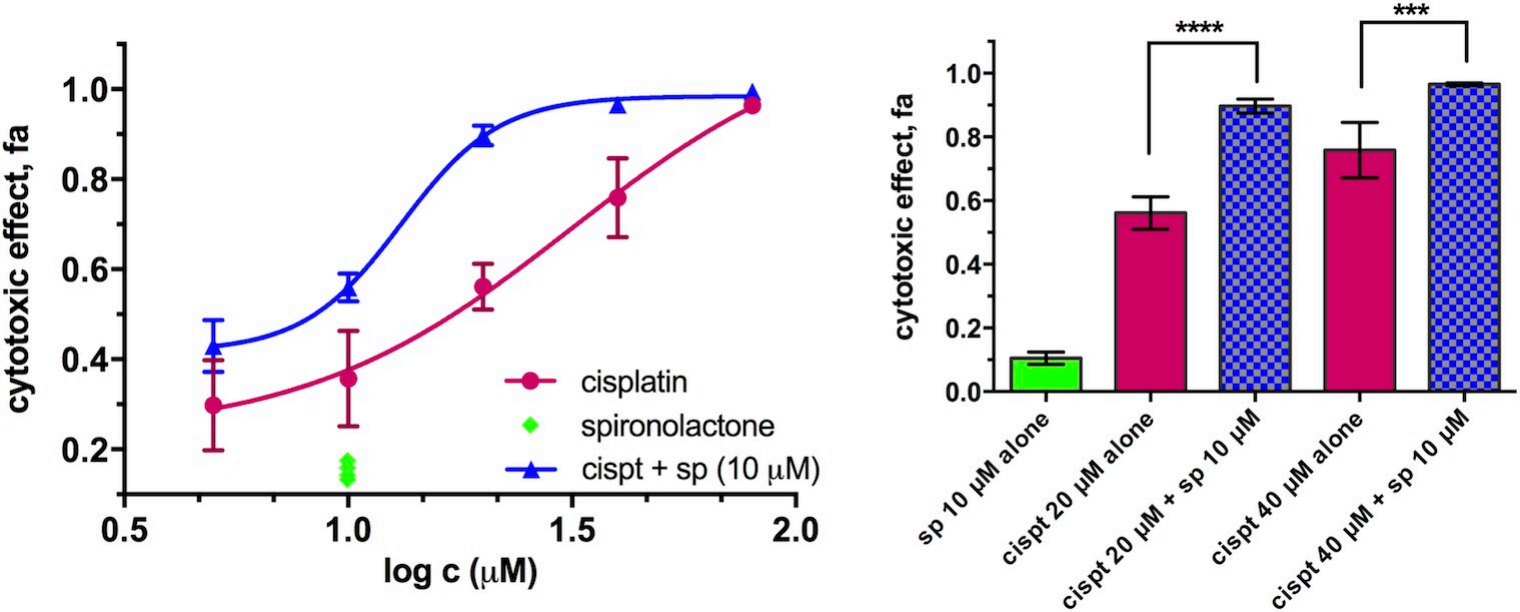
Cytotoxicity of cisplatin alone and in combination with spironolactone in PANC-1 cells after 72 h of exposure. Left: concentration effect curves and Right: bar graphs of selected concentrations. Data is presented as mean ± SD from 4 replicates (combination) or 8 replicates (drugs alone) per concentration (****p < 0.0001, ***p < 0.001, determined by unpaired t test with Welch’s correction). Statistical analysis, curve fitting and graphs are prepared with GraphPad Prism 7.

Spironolactone (see S7 Fig for chemical structure) is inactive at a concentration of 10 μM, but potentiates the cytotoxicity of cisplatin in PANC-1 cells, resulting in a 2.5-fold decrease of the IC_50_ and a 3-fold decrease of IC_90_ values (Fig 8). This supports the work of Coin *et al*. [59] on the repositioning of spironolactone as an adjuvant in platinum-based therapy, particularly for highly chemoresistant pancreatic carcinoma. Further experiments showed that spironolactone can enhance the activity of cisplatin to certain extent also in other cancer cells and in some cases also in non-cancer cell lines (see S13 Fig).

### HCA using automated fluorescence microscopy

A secondary assay, based on HCA was employed in order to validate the synergistic combinations using a different type of readout, as well as to provide further insights into the synergistic interactions. In addition, HCT116 (colorectal carcinoma) cells and the non-cancer cell lines MRC-5 and BJ were included to further investigate the effects of the combinations. Hence, selected compounds were tested alone and in combinations at 4 concentrations and at 3 different exposure times (i.e. 24, 48, 72 h). The images acquired after staining were segmented and analyzed with Cell Profiler software [64,65], and from the parameters extracted from the segmented objects, the following were selected for further analysis: count of cells (N_cells_), mean intensity of PI on the nucleus (Ī_PI_) and correlation of Hoechst and PI intensities in the nucleus (Q_PI/Ho_). N_cells_ and Ī_PI_ for the different compounds and combinations were normalized to the values obtained for the negative controls (cells + 0.1% DMSO) at the respective time point and expressed as a percentage of untreated controls, %N_cells_, or as an intensity ratio, _n_I(PI) = Ī_PI_(drug)/ Ī_PI_(neg.ctrl), respectively.

**Fig 9:**
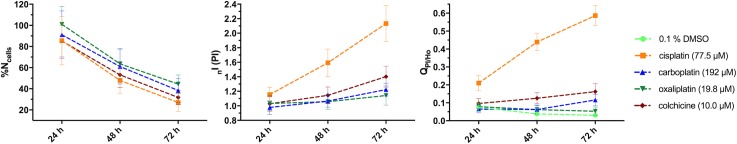
Comparison of %N_cells_, _n_I(PI) and Q_PI/Ho_ obtained over time for PANC-1 cells exposed to Pt drugs and colchicine. Values are acquired as mean ± SD from at least 2 wells/condition and 9 field of view/well. For fluorescent microscopy pictures of PANC-1 and A549 cells after 72 h of exposure to the Pt drugs, see S14 and S15 Figs.

Most of the compounds and combinations affect the cells in a time- and dose-dependent manner, usually with only marginal affects after 24 h (especially in PANC-1 cells). Significant effects on the viability of PANC-1 cells at 24 h of incubation were, however, observed for auranofin. Notably, a decrease of %N_cells_ with the concentration and/or time of exposure was not always accompanied with an increase in Q_PI/Ho_ or Ī_PI_ (a measure for the amount of dead objects in the well). The increase of Ī_PI_ directly corresponds to the cytotoxicity of a drug, whereas a decrease of %N_cells_ could also be due to inhibition of cell division (i.e., cytostatic effect). Cisplatin, for example, demonstrated strong cytotoxic effects at higher concentrations, provoking a significant increase in Ī_PI_ (up to 2 times for PANC-1 and 3.5 times for A549 cells, compared with the control). In certain cases (e.g., carboplatin, oxaliplatin and the control colchicine in PANC-1 cells, oxaliplatin in A549 cells as well as cisplatin in the lowest concentrations tested in both cell lines) no increase or only a marginal increase of _n_I(PI) was observed, despite the N_cells_ being less than 50% of that in the negative control (see Fig 9).

**Fig 10:**
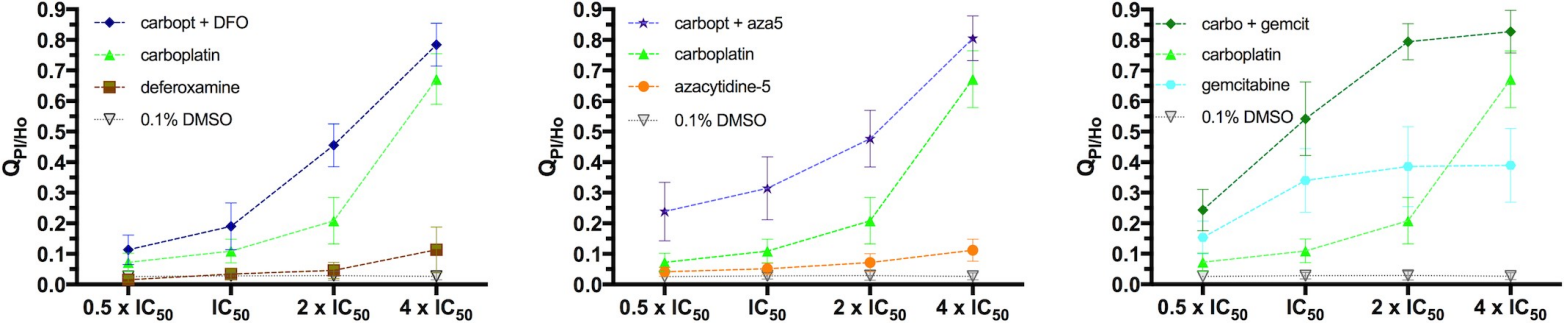
**Synergistic combinations between carboplatin and deferoxamine (left), azacytidine-5 (center) and gemcitabine (right) in A549 cells.** Dose-dependent increase of Q_PI/Ho_ after 72 h of continuous exposure to the drugs alone (in concentrations ranging from ½ x IC_50_ to 4 x IC_50_) and their combinations at a fixed concentration ratio. Values are obtained as mean ± SD from 2 wells/condition and 9 field of view/well.

**Fig 11:**
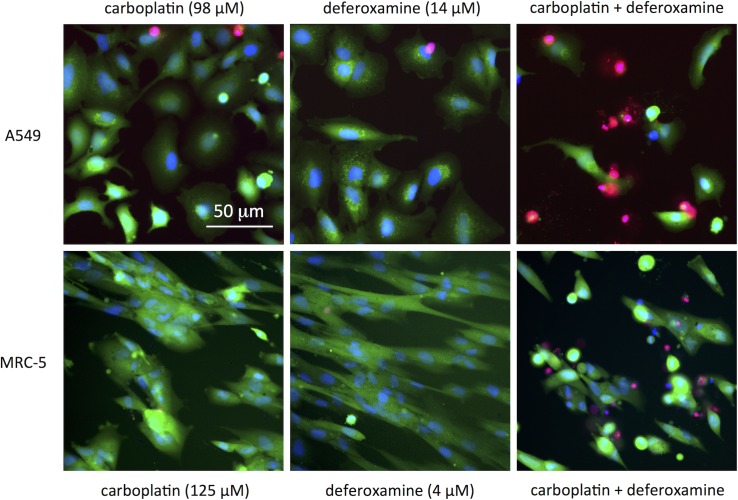
**Images of A549 (top) and MRC-5 (bottom) cells exposed to carboplatin, deferoxamine and their combination** (72 h of continuous exposure). Green channel: Calcein AM (live cells), blue channel: Hoechst (nuclei), red channel: PI (dead cells).

Comparison of the parameters %N_cells_, _n_I(PI) and Q_PI/Ho_ obtained after treatment with the compounds alone with those obtained after using the respective combinations suggested synergistic interactions in most of the cases, more evident at 48 h and especially at 72 h of exposure. An effect on all three parameters was observed in A549 cells for the combinations of carboplatin with deferoxamine and with some antimetabolites (see Fig 10 and S17 Fig, as well as Fig 11, S16–S18 Figs for representative fluorescent microscopy images). Notably, the combination between carboplatin and deferoxamine is less toxic to the non-cancer MRC-5 cells, than to A549 cancer cells (both cell lines from lung origin) at comparable carboplatin concentrations (see Fig 11). However, at higher concentrations synergistic toxicity could be observed also in MRC-5 cells, but not for cell lines from other origins (see S19 Fig).

**Fig 12:**
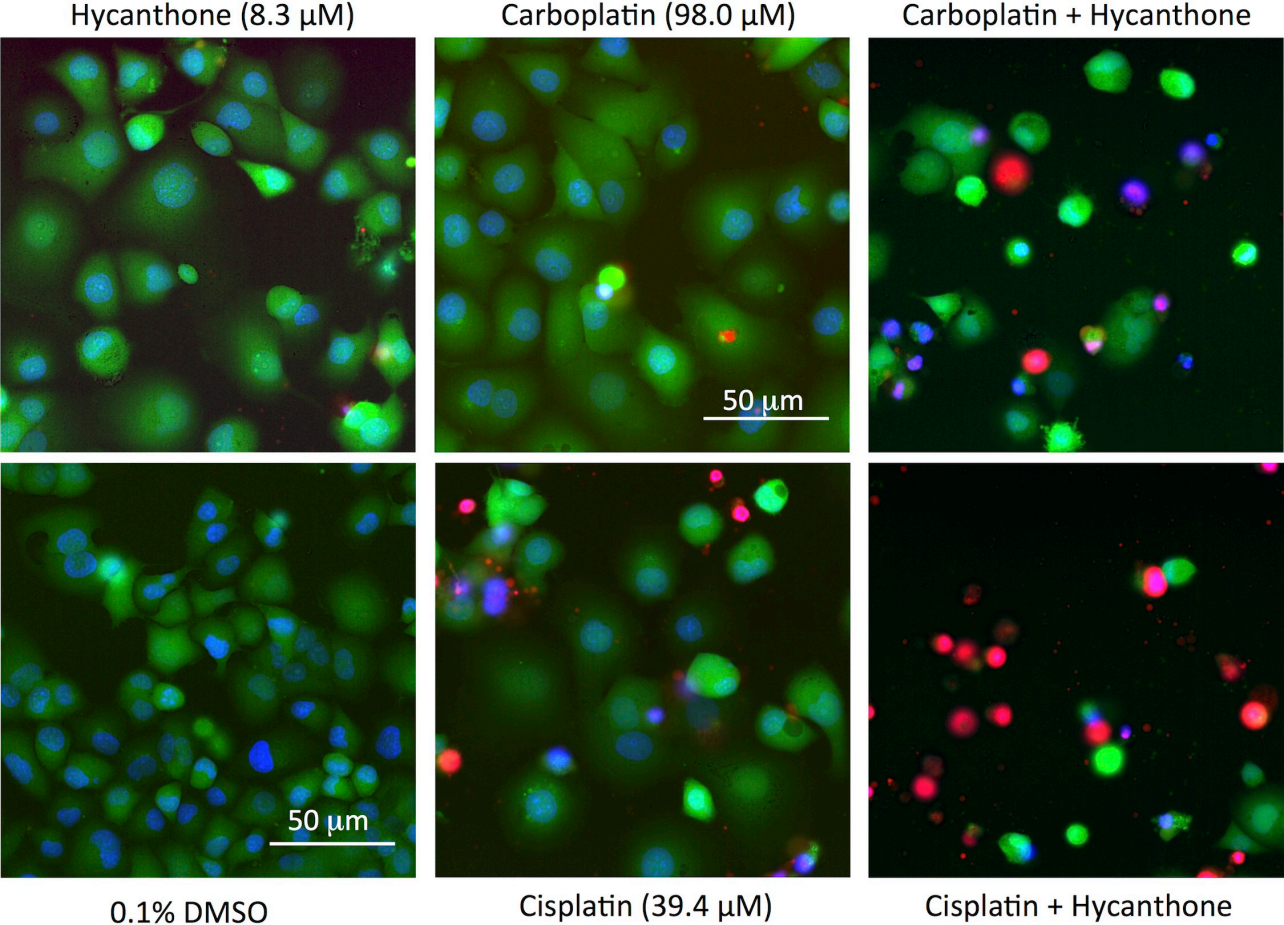
Images of PANC-1 cells exposed to hycanthone, cisplatin, carboplatin and their respective combinations. Untreated controls (cells + 0.1% DMSO) are shown for comparison. Drug exposure time was 72 h. Green channel: Calcein AM (live cells), blue channel: Hoechst (nuclei), red channel: PI (dead cells).

Strong synergistic interactions between hycanthone and cisplatin, and especially carboplatin were observed in PANC-1 cells (Fig 12, S20 and S21 Figs). These findings highlight the potential of these drug combinations in the development of more efficient treatment regiments for pancreatic cancer. Hycanthone seems to synergize with carboplatin and cisplatin also in HCT116 cells and in the higher concentration settings also in BJ and MRC-5 cells (S21 and S22 Figs).

Spironolactone on its own showed no effect on %N_cells_, _n_I(PI) and Q_PI/Ho_ (in accordance with the PrestoBlue-based assays) in PANC-1 cells, whereas its potentiating effect on cisplatin can be appreciated from the increase in Q_PI/Ho_.

Accurate quantitative analysis was not possible for certain compounds (i.e., camptothecine, topotecan, daunorubicin, aminacrine), due to their intrinsic fluorescence, which interferes with at least one of the stains. However, indications for the synergistic combinations between Pt drugs and camptothecines could still be seen. Indeed, significant increases in the cytotoxicity of topotecan in combinations with carboplatin and cisplatin was observed in all cell lines tested (S23 Fig). Despite the demonstrated lack of selective synergism (synergistic only against cancer cells), topotecan-platinum combinations are successfully used in several cancer treatment regimens, and their toxicity profiles are clinically acceptable.

## Conclusions

Currently, there is still no adequate treatment of lung and pancreatic carcinomas, and consequently they remain amongst the most deadly cancers. Combinations, featuring Pt drugs are among the most efficient treatments available, however, survival rates are still very low (5-year survival rates of under 10%), highlighting the necessity for development of new therapeutic options for these malignancies. In an attempt to improve platinum-based chemotherapy of problematic cancers, we have established a cell-based screening assay that identifies compounds, which either potentiate the activity or synergize with Pt drugs. These compounds are identified from a library with FDA approved drugs using a systematic screening approach. As drug combinations and synergy determination can be problematic, screening was followed by hit confirmation and validation procedures, including synergy quantification and HCA.

Several new promising synergistic combinations were discovered that include compounds currently not used as anticancer drugs. The most prominent examples comprise cisplatin/carboplatin+hycanthone, cisplatin/carboplatin+topotecan, cisplatin+vorinostat and cisplatin+spironolactone in pancreatic carcinoma, and carboplatin+deferoxamine in lung cancer. Moreover, our data revealed stronger synergistic interactions between cisplatin/carboplatin and topotecan in PANC-1 cells, compared to A549 cells, suggesting that this combination, currently used in lung cancer treatment regiments, could be applied to pancreatic carcinoma.

Several drugs used to treat diseases other than cancer, e.g pyrvinium pamoate, auranofin, terfenadine and haloprogin, showed strong cytotoxicity on their own and synergistic interactions with Pt drugs during the screens. Poor solubility precluded synergistic quantification, however, these compounds deserve attention as they could advance combination chemotherapy of problematic cancers if appropriate formulations can be developed.

The synergistic combinations identified in this study provide a rational base for the design of new platinum-based treatment regimens, as well as for the development of novel multi-action Pt(IV) prodrugs, which can improve the chemotherapy of lung and pancreatic cancers.

## Materials and methods

### Compounds

Cisplatin, carboplatin and oxaliplatin were purchased from TCI and their identity and purity (> 95%) was confirmed by ^1^H NMR spectroscopy and elemental analysis prior to use. Stock solutions were prepared directly before experiments either in water (carboplatin and oxaliplatin) or in PBS (cisplatin) and filtered through sterile membrane filter (0.22 μM) prior use. The PCL was purchased from Prestwick Chemicals (Washington, DC) and contains 1280 molecules, supplied as 10 mM stock solutions in DMSO. Compounds were stored in the dark, at– 20°C under dry air, using an automated storage system and their chemical integrity was controlled regularly by HPLC-MS.

### Cell lines and culture conditions

A549 (human non-small cell lung adenocarcinoma), PANC-1 (human pancreatic adenocarcinoma), HCT116 (human colorectal carcinoma), BJ (human normal foreskin fibroblasts) and MRC-5 (human normal lung fibroblasts) cell lines were purchased from ATCC and were used between passage numbers 10 and 35. All cell culture media, buffers and reagents were obtained from Gibco Life Technologies. Cells were grown as adherent monolayer cultures in 75 cm^2^ culture flasks (TPP) without antibiotics using the following growth media, supplemented with 10% heat-inactivated fetal bovine serum (FBS, Invitrogen 10101–145): Dulbecco’s Modified Eagle medium/F-12 Nutrient mixture (Ham) (DMEM/F-12 + GlutaMAX, 31331–028) for A549 cells, Dulbecco's Modified Eagle's Medium (DMEM, high glucose, GlutaMAX, Pyruvate, 31966–021) for PANC-1 cells, McCoy5a Medium (McCoy5a, GlutaMAX, 36600–021) for HCT116 cells and Eagles’s Minimum Essential Medium (EMEM, low glucose, L-Glutamine, 11095–080) for BJ and MRC-5 cells. Cultures were maintained at 37°C in a humidified CO_2_ incubator.

Cells were subcultured 2 to 3 times per week. Briefly, the cells were harvested with Trypsin 0.05%-EDTA (Life Technologies 25300062) and diluted with growth medium (1:3 to 1:5 for PANC-1, BJ and MRC-5 cells, 1:5 to 1:20 for A549 and HCT116 cells). Cells used for the assays were harvested from culture when the level of confluence was between 60% and 80%, while cell viability was > 90%.

### Primary HTS assay

The compounds from the PCL were dispensed into sterile, basic flat-bottom, culture-treated, transparent, barcoded 384-well plates (Corning 3701), using an acoustic liquid handler Echo 550 (Labcyte Inc. Sunnyvale, CA). Each drug was added once (one well per compound), volume 30 nl, yielding a final concentration of the compound of 10 μM and final DMSO concentration of 0.1%. The first two columns of every plate were used as negative control (no PCL drug added) and filled with an equivalent volume of DMSO (30 nl/well) while the last two columns were filled with doxorubicin (10 μM with DMSO, 0.1%) for the positive control; the entire PCL can be processed on four such plates (see ref.[26]). Cells were harvested by tripsinization and seeded in complete growth medium into the plates in volumes of 25 μl/well using a multi-drop dispenser (Thermo Scientiffic Multidrop Combi) at medium speed. A seeding density of 2000 cells/well was used for both A549 and PANC-1 cells to ensure exponential growth of untreated controls throughout the experiment and a satisfactory quality of the assay. Next, Pt drugs (in concentration corresponding to their IC_50_ or IC_20_) diluted in medium were added to each well of the PCL-Pt combination plates in volumes of 5 μl/well using a multi-drop dispenser. In the PCL-only plates, equivalent amounts of PBS (respectively water) diluted in medium were added also to all the wells. After a post-plating incubation step at room temperature for 20 min, which ensures a more homogeneous repartition of the cells at the bottom of the wells, plates were transferred to an incubator (37°C, 5% CO_2,_ saturated moist atmosphere) for 72 h (PANC-1 cells) or 50 h (A549 cells). Subsequently, 3 μL of PrestoBlue reagent (Life Technologies, Switzerland) was added to each well and the plates were returned to the cell incubator for 1 h. The fluorescence intensity (bottom-read) was measured using a multiwell plate reader (Tecan Infinite F500) at excitation 560/10 nm, emission 590/10 nm and a fixed gain. The experimental protocol for the screen is summarized in Table 1 and S3 Fig. The screening was performed for each cell line with the full PCL alone or in combination with each of the Pt drugs, tested in two final concentrations; all conditions were assayed in duplicate.

#### Data analysis

The BSF in-house Laboratory Information Management System (LIMS) was used for basic data processing, management, visualization and statistical validation. Data was normalized according to the fluorescence signals of control wells located in the first two and last two columns of every plate and presented as HTS scores, where a score of 0 was assigned to the average fluorescence intensity of the negative control wells and 1 to that of the positive control wells [26]. In the case of the PCL-Pt combination plates, calculation of the scores is based on normalization to the effect of the Pt drug alone as the respective Pt drug (at fixed concentration) is added to every well (including the controls). For each screen plate, HTS scores were considered only when their value is higher than the average of the negative controls + 3 x SD. HTS scores were calculated as mean ± SD from two replicates (every screen plate was ran in duplicate). A higher mean score of a PCL compound in combination with certain Pt drug, compared to the mean score of the PCL compound alone (Mean_PCL+Pt_ + SD_PCL+Pt_ > Mean_PCL_—SD_PCL_) suggests more than cumulative cytotoxicity (i.e. synergistic); such drug combinations were marked as ‘hit-combinations’ (HCs).

Assay quality was assessed by calculation of the screening window coefficient (Z’-factor) for every screen plate. The Z’-factor was determined according to the formula: Z’ = 1–3(SD_pos_ + SD_neg_)/|Av_pos_−Av_neg_|, where SD and Av are the standard deviation and the average, respectively, of the fluorescence signals of the negative and positive control wells [66]. For the combination plates (PCL compounds plus Pt drugs at their IC_50_), a narrower dynamic range and separation band were observed as a result from the lower fluorescence of the negative controls, a consequence of the decreased cell metabolism and viability, caused directly by the Pt drug. The quality of the assay was considered sufficient when Z’ >0.4–0.5

#### Confirmation screening

The PCL compounds identified in HCs during the primary screening assay were dispensed (cherry picked) into 384-well plates using the acoustic liquid handler Echo 550 (Labcyte Inc. Sunnyvale, CA). Library compounds were added in volumes of 30 nl or 2.5 nl (for compounds with scores > 0.85 in the primary screen) to yield final concentrations of 10 μM or 0.83 μM, respectively. The plate layout was redesigned to accommodate the lower number of samples and avoid any edge effect (no controls or compounds in the edge wells). Cell seeding, addition of Pt drugs (or PBS/water), incubation, cell viability determination and data evaluation were as described for the primary screening. All conditions were assayed in duplicate.

### Combination studies and synergy quantification

Confirmed HCs were further evaluated using the CI method of Chou and Talalay with a diagonal constant ratio experimental design [31,32]. Two-fold serial dilutions were performed for each drug alone and for their mixtures at a fixed concentration ratio (e.g. at (IC_50_)_Pt drug_/(IC_50_)_PCL compd_ ratio) to yield 5 concentrations ranging from ¼ x IC_50_ to 4 x IC_50_ (see S24 Fig for the plate layout). PCL compounds were dispensed into 384-well plates using the Echo 550 acoustic liquid handler (Labcyte Inc. Sunnyvale, CA) before seeding the cells. Pt drugs were added either also by the acoustic liquid handler (carboplatin, oxaliplatin) before cell seeding or manually (cisplatin and in some cases oxaliplatin) after cell seeding in order to minimize interactions with the DMSO solvent used in the PCL compounds stocks (see S2 Fig). Cell seeding (2000 cells/well for PANC-1 cells and 1200 cells/well for A549 cells), incubation (72 h for both cell lines) and cell viability determination were accomplished as described for the screening assays. Data was normalized to the controls (20 μM Doxorubicin.HCl and 0.2% DMSO, respectively) and presented as fraction of effect affected (fa). All compound concentrations and combinations were tested in 4 replicates (i.e. 2 wells/plate, every plate in duplicate) and mean fa values and their standard deviations were calculated. Combination effects analysis and CI calculation were performed using CompuSyn software [62]. Linear regression was used to fit the logarithmic form of the median effect equation to the experimental data for each drug and their combinations, allowing the potency, D_m_ (corresponding to the IC_50_), and the shape (sigmoidicity) of the dose-response curve, m, to be determined. The linear correlation coefficient, r, was determined as conformity for goodness of fit and r > 0.85 for both single drugs and combinations was required for a successful analysis. The D_m_ and m values for each drug and their combinations were used to calculate CI as a quantitative measure of the drug interactions in terms of synergism (CI < 1), additive effect (CI = 1) and antagonism (CI > 1) for a given endpoint of the effect measurement. Sequential deletion analysis (S.D.A.) was used as a measure of the variability of the CI values at presented effect levels. Mean ± 95% confidence intervals were calculated at the specified effect levels after an iterative sequential deletion of one concentration of a drug at a time for repetitive CI calculations [31,62]. Combinations with carboplatin in PANC-1 cells were studied at non-equipotency fixed ratios, due to its low activity (IC_50_ ~ 180 μM) towards PANC-1 cells; the highest concentration of carboplatin used (192 μM) resulted in cell viability inhibition, fa = 0.53. Nevertheless, conformity of analysis for carboplatin and its combinations in PANC-1 cells was good (r > 0.9).

Additional combination experiments with fixed concentrations (e.g., 0.1, 1 or 10 μM) of some PCL compounds (corticosteroids, spironolactone) and the Pt drugs in concentrations, ranging from ¼ x IC_50_ to 4 x IC_50_, were also performed. Evaluation of the combination effects in certain cases was assessed by the fractional product method of Webb [63,67]. The cell survival fractions (fu = 1—fa) of the drugs alone were used to calculate the expected additive effects as a product of the individual fractional effects (fu_A+B_^calc^ = fu_A_ x fu_B_) for specific concentrations. A synergistic interaction was assumed when 0.7 x fu_A+B_^calc^—Mean (fu_A+B_^det^) ≥ 3 x SD (fu_A+B_^det^); when Mean (fu_A+B_^det^)– 1.3 x fu_A+B_^calc^ ≥ 3 x SD (fu_A+B_^det^), the combination was considered as antagonistic.

### HCA using automated fluorescence microscopy assays

Experiments were performed using sterile, black/clear tissue culture treated, 384-well plates with flat bottom (BD Falcon Imaging plates 353962). Selected PCL and Pt drugs (alone and in combinations at four different concentrations, two wells per condition) were added using the Echo acoustic dispenser (PCL compounds) or manually after cell seeding (Pt drugs). Cells were seeded (in medium and at volume of 30 μl/well) into the plates using a multidrop dispenser, medium speed at density of 2100 cells/well for PANC-1 cells, 1100 cells/well for A549 cells, 2000 cells/well for BJ and MRC-5 cells and 3000 cells/well for HCT116 cells. The plates were then incubated at 37°C and 5% CO_2_ for 24, 48 and 72 h prior staining. The staining solution was prepared freshly by diluting DMSO stocks of Calcein AM (Invitrogen C3100MP) and Hoechst 33342 (Sigma B2261), and aqueous stock solution of propidium iodide (PI, Fluka 81845) in PBS, and added to the cells manually at volumes of 10 μl/well. The final concentrations of the dyes were as follows: 2 μg/ml for Hoechst, 0.16 μg/ml for Calcein AM and 6.66 μg/ml for PI in the case of A549, BJ and MRC-5 cells and double diluted for PANC-1 and HCT116 cells. Plates were then returned to the incubator for another 30 minutes for full staining. Microscopy reading was performed in an environmental chamber (37°C, 5% CO_2_) using a GE Healthcare IN Cell Analyzer 2200. Imaging was performed with a 20X/0.75 objective. For each well 9 field of view were acquired, each with 3 channels. Hoechst, PI and Calcein AM signals were imaged using the filter combinations 390/18-432/48, 542/27-597/45, 475/28-512/23, respectively. Exposure times were set respectively at 80, 5 and 100 ms for PANC-1 cells, 100, 5 and 100 ms for A549 cells, 80, 50, and 8 ms for BJ and MRC-5 cells and 60, 10 and 40 ms for HCT116 cells. Images were analyzed using Cell Profiler and Cell Profiler analyst software [64,65].

## Supporting information

S1 Fig Time/concentration dependent cytotoxicity of the Pt drugs and doxorubicin hydrochloride in PANC-1 and A549 cells. Experiments were performed in 96-well format; cell viability was determined by the PrestoBlue fluorescence assay. A) Dose response curves of PANC-1 cells after continuous drug exposure for 48 h, 72 h and 96 h (cell seeding density at 7500, 6000 and 5000 cells/well, respectively). B) Dose response curves of A549 cells after continuous drug exposure for 24 h, 48 h, 72 h and 96 h (cell seeding density at 10000, 6000, 4000 and 2500 cells/well, respectively). C) 50% inhibitory concentrations (IC_50_) determined for the investigated compounds in PANC-1 (left) and A549 (right) cells after different exposure times; IC_50_ values are plotted as mean ± SDs from at least two independent experiments.
(PDF)

S2 Fig Effect of DMSO on the cytotoxicity of Pt(II) complexes. (PDF)

S3 Fig Graphic summary of the screening experimental protocol. I) Addition of PCL compounds (10 μM, 1 well/compound, 320 compounds/screening plate), positive control (10 μM Doxorubicin.HCl, last two columns of every screening plate) in green and negative control (0.1% DMSO, first two columns of every screening plate) in light blue; II) Addition of cell suspension, followed by addition of Pt drugs in medium (PCL+Pt plates) or PBS/water in medium (PCL only plates) in every well of the respective plate; III) Cell viability determination by means of the Presto Blue assay after the respective drug exposure times; IV) Data processing, management and statistical validation; identification of HCs. All conditions were assayed in duplicate.
(TIFF)

S4 Fig **HCs (above the additive line) in A) PANC-1 and B) A549 cells identified during the primary screening.** HTS Scores from the combination (i.e., PCL+Pt drugs) plates are plotted vs. the scores obtained from the PCL alone plates. Scores are given as mean from 2 replicates (2 wells/drug, respectively drug combination).
(TIFF)

S5 Fig HTS scores of daunorubicin. **HCl alone and in combination with cisplatin or carboplatin** obtained at the confirmation screening low concentration setting in PANC-1 cells. Data is presented as mean ± SD from 2 replicates (**p < 0.01, determined by unpaired t test with Welch’s correction using GraphPad Prism 7). Chemical structures (right).
(TIFF)

S6 Fig HTS scores of chosen antimetabolites (10 μM) alone and in combination with cisplatin (7 μM) obtained at the confirmation screening in A549 cells. Data is presented as mean ± SD from 2 replicates (**p < 0.01 and *p < 0.05, determined by multiple (unpaired) t-tests with GraphPad Prism 7). Chemical structures (right).
(TIFF)

S7 Fig Chemical formula of the antihypertensive drug spironolactone. (TIF)

S8 Fig Synergistic combinations of carboplatin in PANC-1 cells. Left: Fa-CI plot of Chou-Talalay for carboplatin + topotecan (1 : 0.08; in blue), carboplatin + aminacrine (1 : 0.03; in red) and carboplatin + hycanthone (1 : 0.10; in green) combinations (72 h of exposure). Error bars represent 95% confidence intervals of the CI variability at the presented effect levels, as determined by S.D.A. Right: Chemical formulas.
(TIFF)

S9 Fig Synergistic combination of cisplatin and vorinostat (1 : 0.75) in PANC-1 cells. Left: classical isobologram at 0.5, 0.7 and 0.9 effect level (IC_50_, IC_75_ and IC_90_ concentrations, respectively). Markers for the actual combination points are pattern filled. Right: Chemical formulas.
(TIFF)

S10 Fig Synergistic combinations between cisplatin and antimetabolites in A549 cells. Classical isobolograms at 0.5, 0.7 and 0.9 effect level (IC_50_, IC_75_ and IC_90_ concentrations, respectively) for the combinations of cisplatin with azacytidine-5, 1 : 3.72 (left) and cisplatin with gemcitabine, 1 : 0.01 (right). Markers for the actual combination points are pattern filled.
(TIFF)

S11 Fig Concentration effect curves of oxaliplatin, alone and in combination with corticosteroids: Prednicarbate (at fixed concentration of 0.1 μM) and flumethasone (at fixed concentration of 1.6 μM) in A549 cells after 72 h of exposure. Curves fitting and graphs are prepared with GraphPad Prism 7.
(TIFF)

S12 Fig Combination effects evaluated by the fractional product method of Webb. A-D): additive to antagonistic interactions between Pt drugs and corticosteroids or paclitaxel in A549 cells; E-F): synergistic interactions between cisplatin and vorinostat, and carboplatin and topotecan in PANC-1 cells. Cytotoxicity of the drugs alone and in combination at different concentrations after 72 h of continuous exposure are expressed as cell survival fractions (fu). Data is shown as mean ± SD from 4–8 replicates per concentration. Expected additive interactions for every drug combination are presented as patterned filled graph bars; error bars in this case mark the region of additivity (between 0.7 x fu_A+B_^calc^ and 1.3 x fu_A+B_^calc^). A, antagonistic drug interaction (**A, denoted when: Mean (fu_A+B_^det^)– 1.3 x fu_A+B_^calc^ ≥ 3 x SD (fu_A+B_^det^); *A, denoted when: Mean (fu_A+B_^det^)– 1.3 x fu_A+B_^calc^ ≥ SD (fu_A+B_^det^)); S, synergistic drug interaction (**S, denoted when: 0.7 x fu_A+B_^calc^—Mean (fu_A+B_^det^) ≥ 3 x SD (fu_A+B_^det^); *S, denoted when: (0.7 x fu_A+B_^calc^—Mean (fu_A+B_^det^) ≥ SD (fu_A+B_^det^))
(TIFF)

S13 Fig Cytotoxicity of cisplatin alone and in combination with spironolactone in PANC-1, HCT116, MRC-5 and BJ cells. Cell viability was assessed by the PrestoBlue fluorescent assay after 72 h of continuous drugs exposure. Data is presented as mean ± SD from 2–4 replicates (combination) or 4–8 replicates (drugs alone) per concentration (****p < 0.0001, ***p < 0.001, **p < 0.01 and *p < 0.05, determined by unpaired t test with Welch’s correction). Statistical analysis, curve fitting and graphs are prepared with GraphPad Prism 7.
(TIFF)

S14 Fig Images of PANC-1 cells after 72 h of exposure to the Pt drugs. Untreated controls (cells + 0.1% DMSO) are shown for comparison. Green channel: Calcein AM (live cells), blue channel: Hoechst (nuclei), red channel: Propidium Iodide (dead cells).
(TIFF)

S15 Fig Images of A549 cells after 72 h of exposure to the Pt drugs. Untreated controls (cells + 0.1% DMSO) are shown for comparison. Green channel: Calcein AM (live cells), blue channel: Hoechst (nuclei), red channel: Propidium Iodide (dead cells).
(TIFF)

S16 Fig Images of A549 cells exposed to carboplatin, gemcitabine, azacytidine-5 and the respective combinations (72 h of continuous exposure). Green channel: Calcein AM (live cells), blue channel: Hoechst (nuclei), red channel: PI (dead cells).
(TIFF)

S17 Fig **Synergistic combinations between carboplatin and azacytidine-5 (left, center) or gemcitabine (right) in A549 cells.** Time dependent increase of nI(PI) (left) and QPI/Ho (center, right) after treatment with the drugs alone and in combinations. Values are obtained as mean ± SD from at least 2 wells/condition and 9 field of view/well.
(TIFF)

S18 Fig Images of A549 cells exposed to the combination of carboplatin (98.0 μM) and azacytidine-5 (31.6 μM). Untreated controls (cells + 0.1% DMSO) are shown for comparison. Green channel: Calcein AM (live cells), blue channel: Hoechst (nuclei), red channel: PI (dead cells).
(TIFF)

S19 Fig Comparison of Q_PI/Ho_ obtained for BJ, MRC-5, A549 and HCT116 cells exposed to carboplatin, deferoxamine and their combination for 72 h. Values are acquired as mean ± SD from at least 2 wells/condition and 9 field of view/well. Carboplatin concentrations at which the drug has similar potency in the different cell lines were chosen for the experiment in order to facilitate comparisons.
(TIFF)

S20 Fig Synergistic combinations between hycanthone and cisplatin or carboplatin in PANC-1 cells. Time dependent increase of nI(PI) (left) and QPI/Ho (center) after treatment with the drugs alone (hycanthone, 15.8 μM; cisplatin, 77.5 μM and carboplatin, 192 μM) and in combinations. Right: dose-dependent increase of QPI/Ho after 72 h exposure to the drugs alone (in concentrations ranging from ½ x IC50 to 4 x IC50) and their combinations at a fixed concentration ratio. Values are obtained as mean ± SD from at least 2 wells/condition and 9 field of view/well.
(TIFF)

S21 Fig **Images of PANC-1 (top) and HCT116 (bottom) cells after 72 h of exposure to hycanthone, carboplatin and their combination.** Green channel: Calcein AM (live cells), blue channel: Hoechst (nuclei), red channel: Propidium Iodide (dead cells).
(TIFF)

S22 Fig **Comparison of QPI/Ho obtained for PANC-1, MRC-5, BJ and HCT116 cells exposed to carboplatin, hycanthone and their combination for 72 h.** Values are acquired as mean ± SD from at least 2 wells/condition and 9 field of view/well.
(TIFF)

S23 Fig **Images of PANC-1 (top) and MRC-5 cells (bottom) after 72 h of exposure to carboplatin, topotecan and their combination.** Green channel: Calcein AM (live cells), blue channel: Hoechst (nuclei), red channel: Propidium Iodide (dead cells).
(TIFF)

S24 Fig Schematic representation of the plate layout used in the drug combinations dose response studies. First and last two rows and columns are drug-free (cells only), columns with ‘+’ are the positive controls (20 μM Doxorubicin.HCl, 0.2% DMSO), rows ‘0’ are the negative controls (0.2% DMSO); Pt(1–5): the respective Pt drug in five concentrations, Lx(1–5): library compound in five concentrations, Cx(1–5): combination of the Pt and the PCL compound at the same concentrations (i.e. ¼ IC50, ½ IC50, IC50, 2 IC50 and 4 IC50 of the respective compounds). DMSO is added to all wells to reach the concentration of the control wells. Total volume per well after seeding the cells is 30 μl. Eight drug combinations can be tested on one such plate. Every plate is prepared in duplicate.
(TIFF)
