# Supplementary figures and images for: Screening-based approach to discover effective platinum-based chemotherapies for cancers with poor prognosis
Source: PLoS One. 2019 Jan 29;14(1):e0211268. doi: 10.1371/journal.pone.0211268 (PMC6350982; doi:10.1371/journal.pone.0211268)

**A**

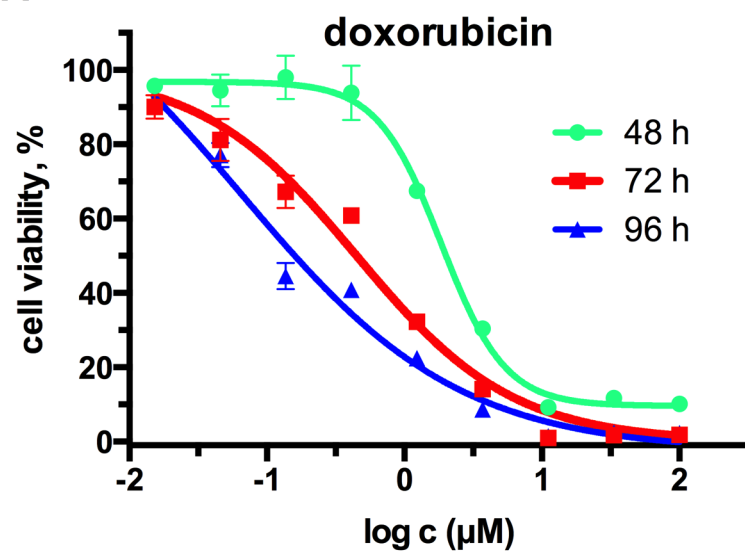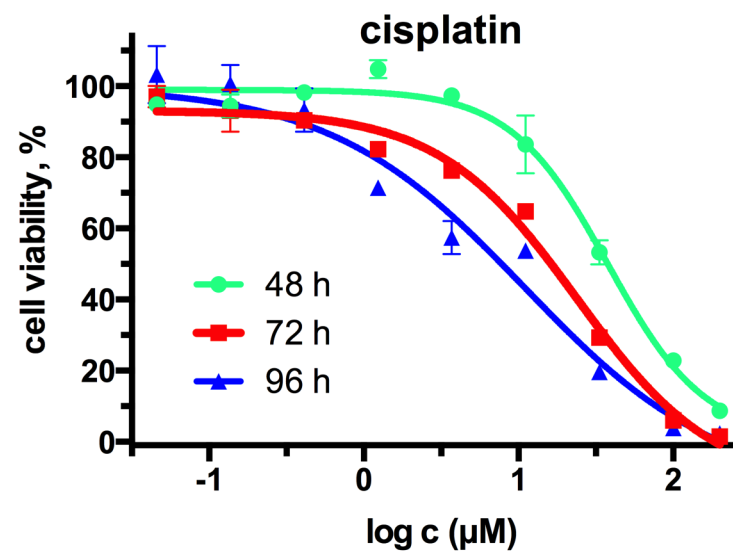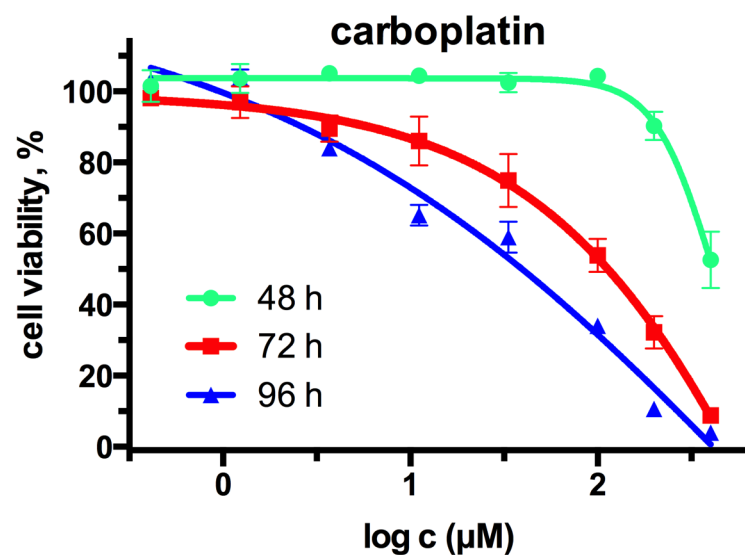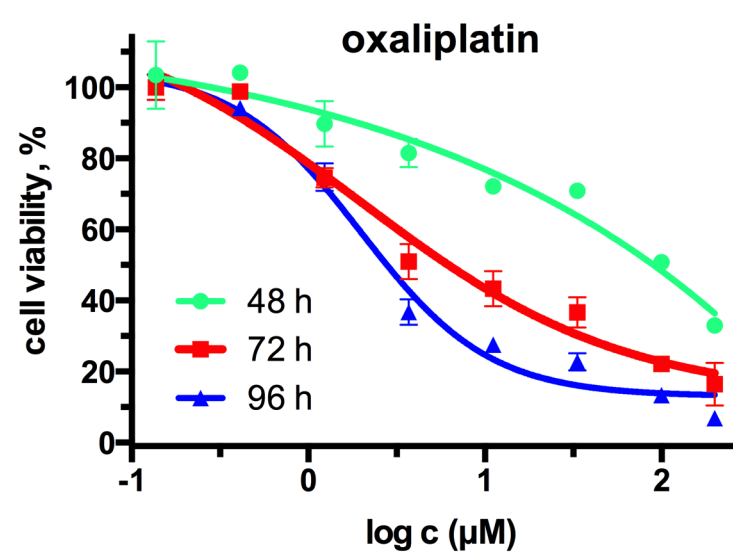

**B**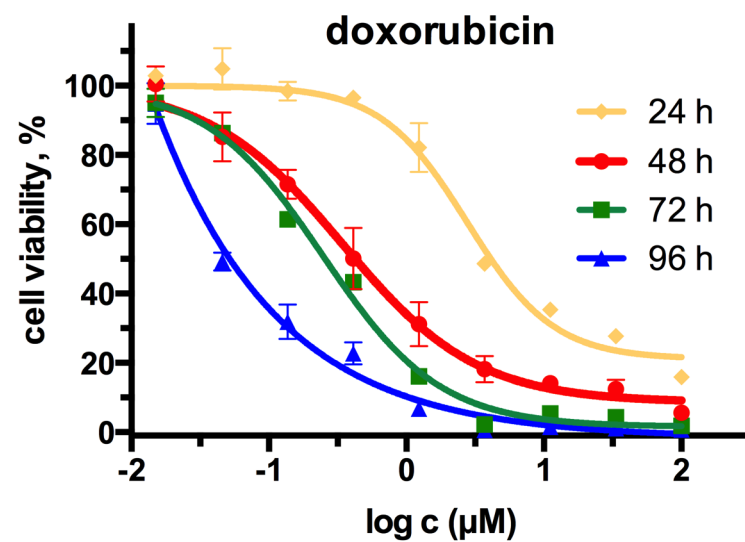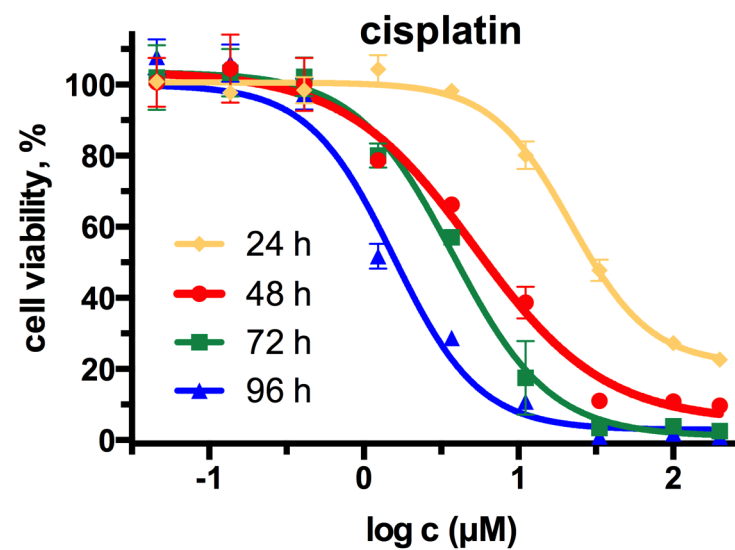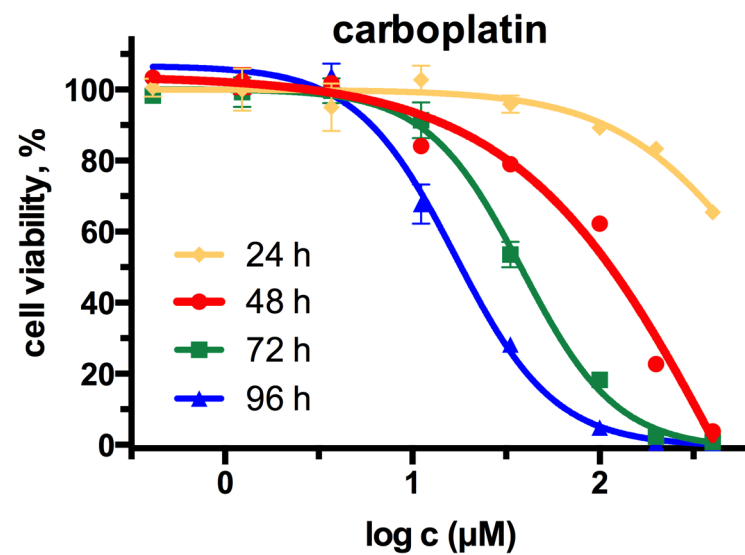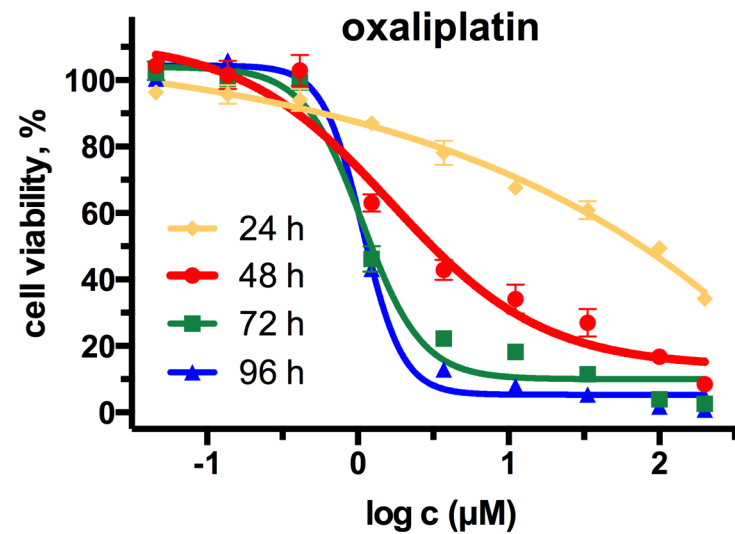

C

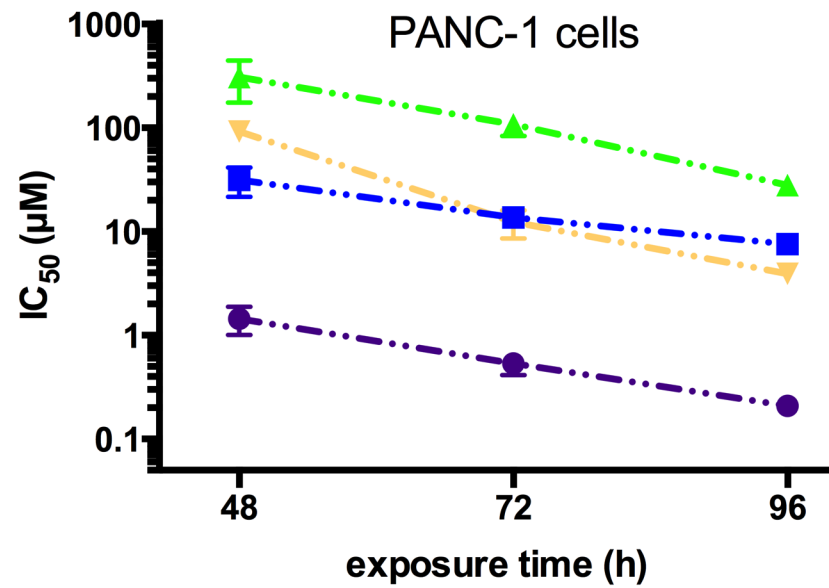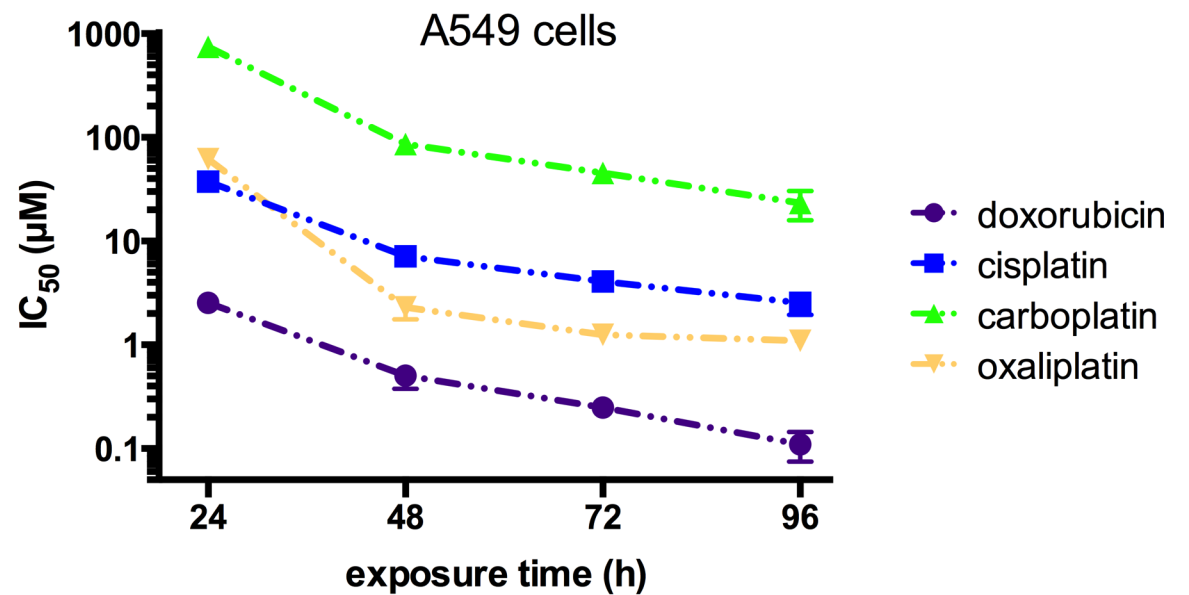

Supplement: S1 Fig — Experiments were performed in 96-well format; cell viability was determined by the PrestoBlue fluorescence assay. A) Dose response curves of PANC-1 cells after continuous drug exposure for 48 h, 72 h and 96 h (cell seeding density at 7500, 6000 and 5000 cells/well, respectively). B) Dose response curves of A549 cells after continuous drug exposure for 24 h, 48 h, 72 h and 96 h (cell seeding density at 10000, 6000, 4000 and 2500 cells/well, respectively). C) 50% inhibitory concentrations (IC50) determined for the investigated compounds in PANC-1 (left) and A549 (right) cells after different exposure times; IC50 values are plotted as mean ± SDs from at least two independent experiments. (PDF) [file pone.0211268.s001.pdf]

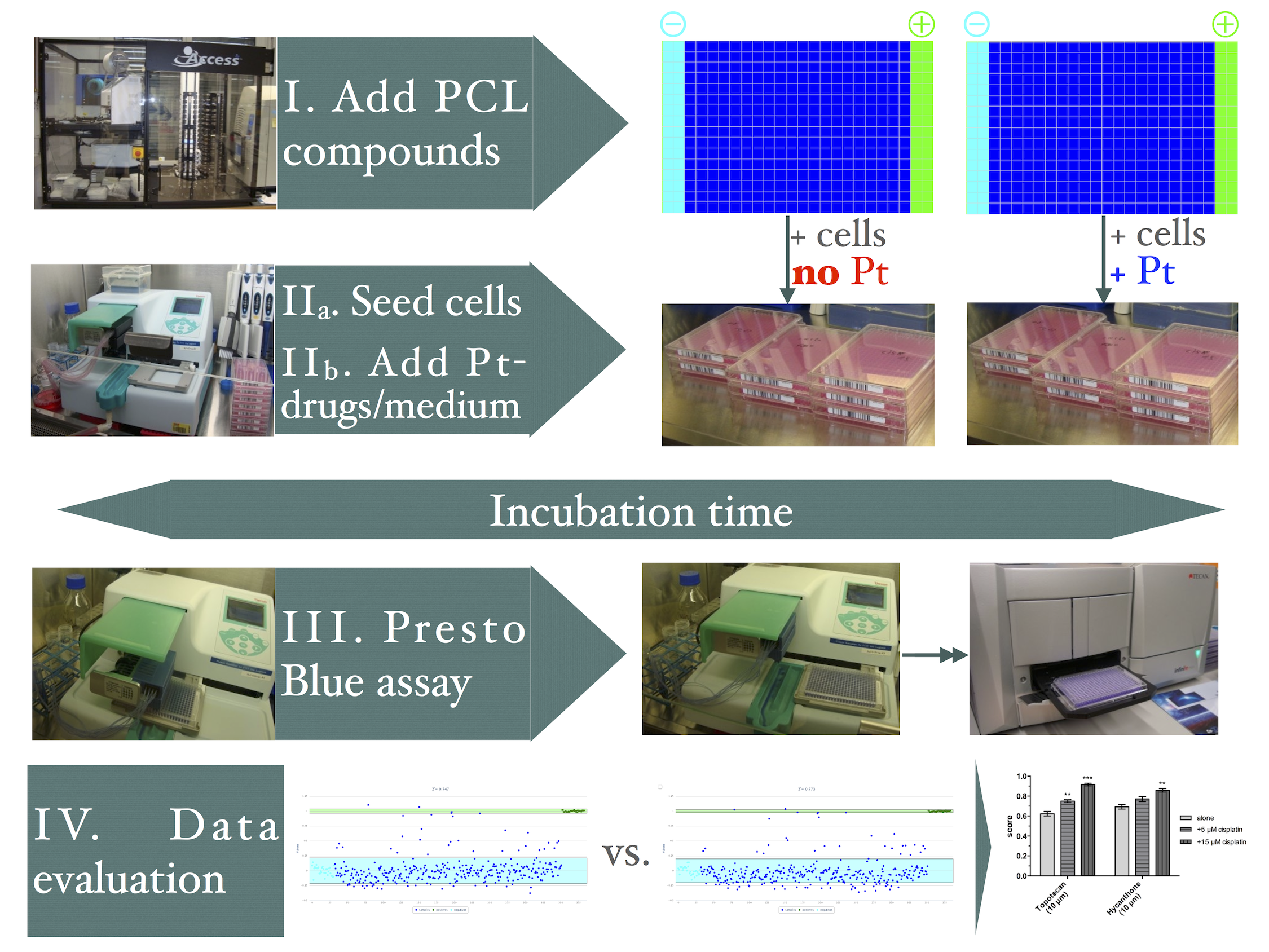

Supplement: S3 Fig — I) Addition of PCL compounds (10 μM, 1 well/compound, 320 compounds/screening plate), positive control (10 μM Doxorubicin.HCl, last two columns of every screening plate) in green and negative control (0.1% DMSO, first two columns of every screening plate) in light blue; II) Addition of cell suspension, followed by addition of Pt drugs in medium (PCL+Pt plates) or PBS/water in medium (PCL only plates) in every well of the respective plate; III) Cell viability determination by means of the Presto Blue assay after the respective drug exposure times; IV) Data processing, management and statistical validation; identification of HCs. All conditions were assayed in duplicate. (TIFF) [file pone.0211268.s003.tiff]

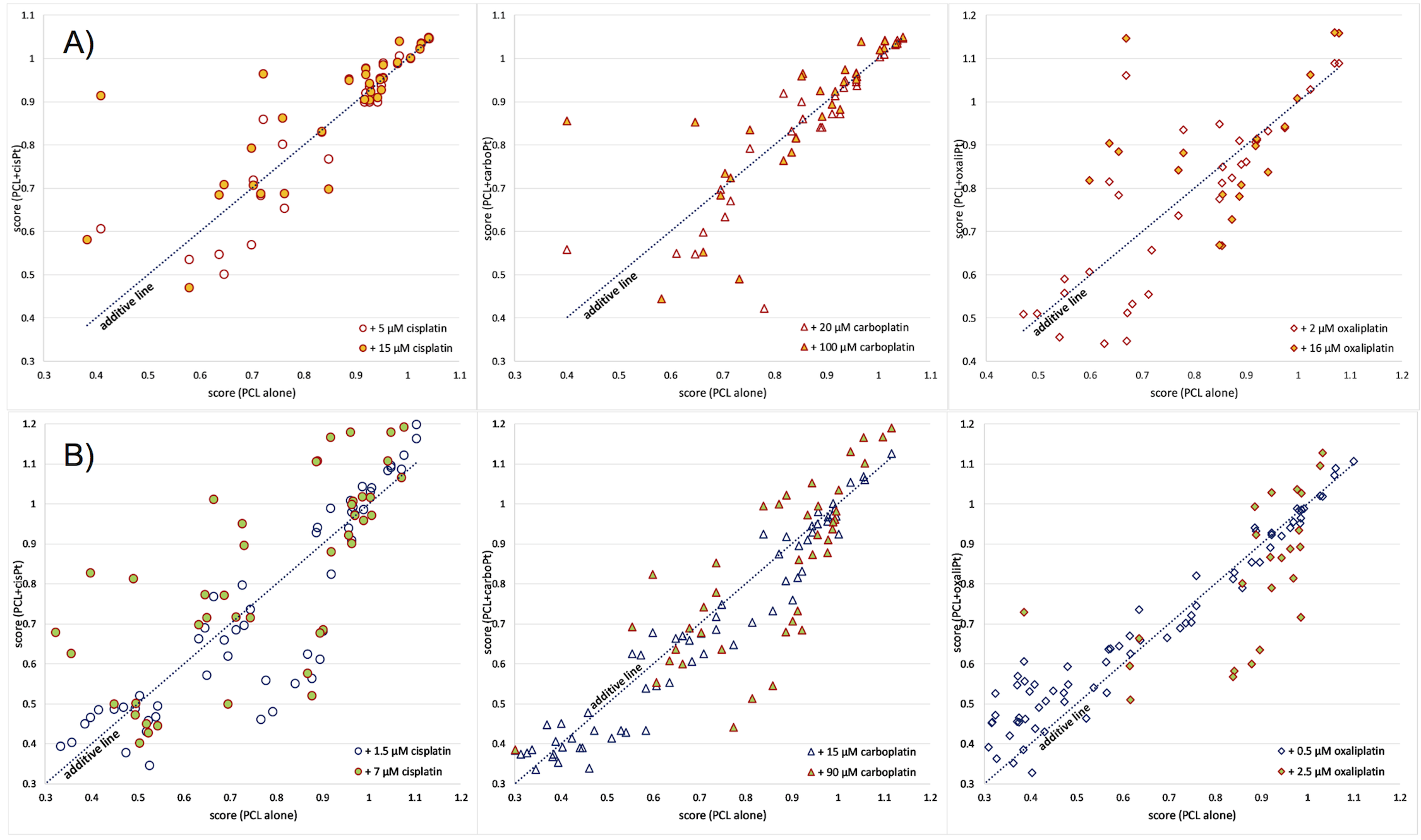

Supplement: S4 Fig — HCs (above the additive line) in A) PANC-1 and B) A549 cells identified during the primary screening. HTS Scores from the combination (i.e., PCL+Pt drugs) plates are plotted vs. the scores obtained from the PCL alone plates. Scores are given as mean from 2 replicates (2 wells/drug, respectively drug combination). (TIFF) [file pone.0211268.s004.tiff]

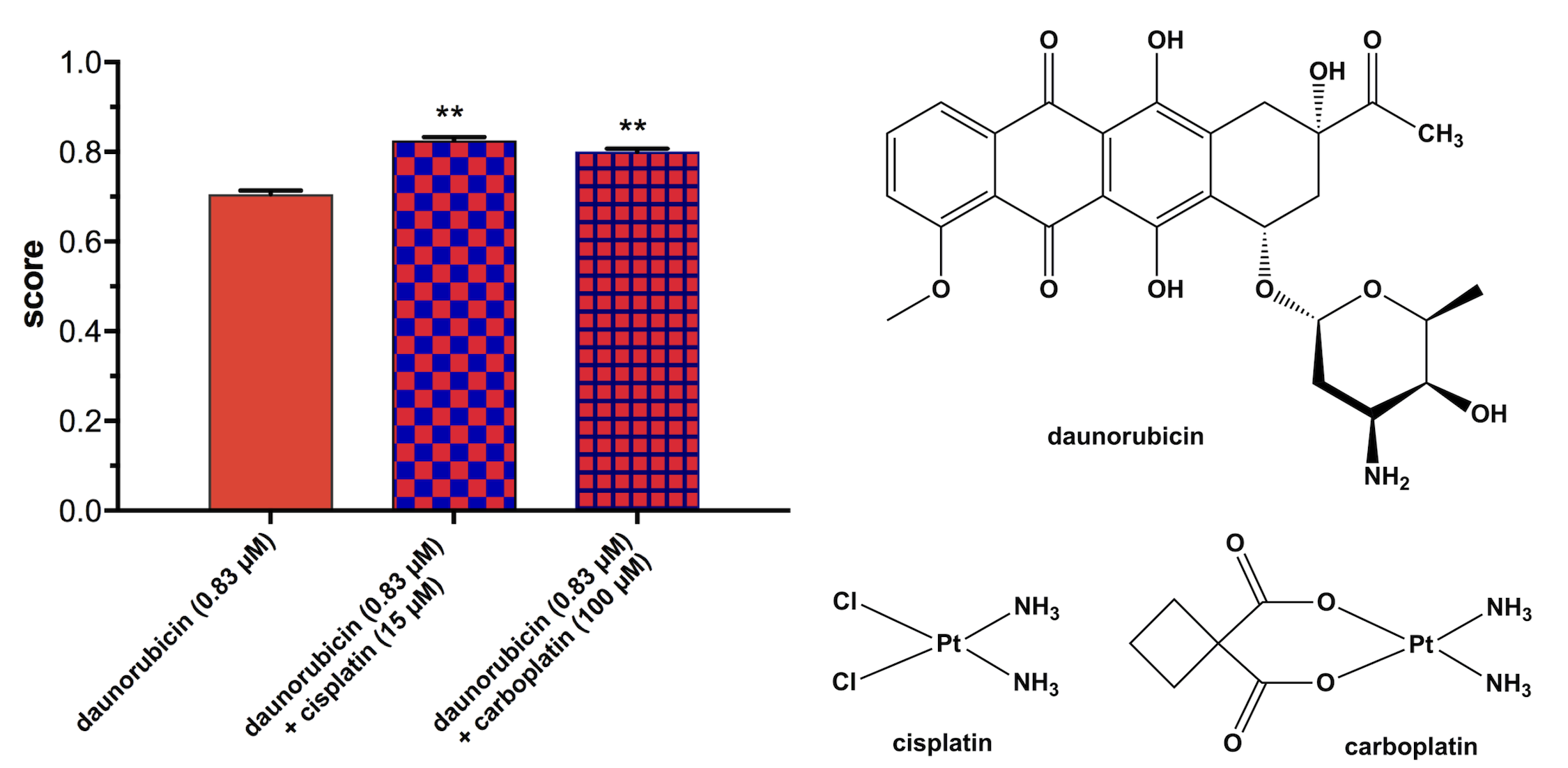

Supplement: S5 Fig — HCl alone and in combination with cisplatin or carboplatin obtained at the confirmation screening low concentration setting in PANC-1 cells. Data is presented as mean ± SD from 2 replicates (**p < 0.01, determined by unpaired t test with Welch’s correction using GraphPad Prism 7). Chemical structures (right). (TIFF) [file pone.0211268.s005.tiff]

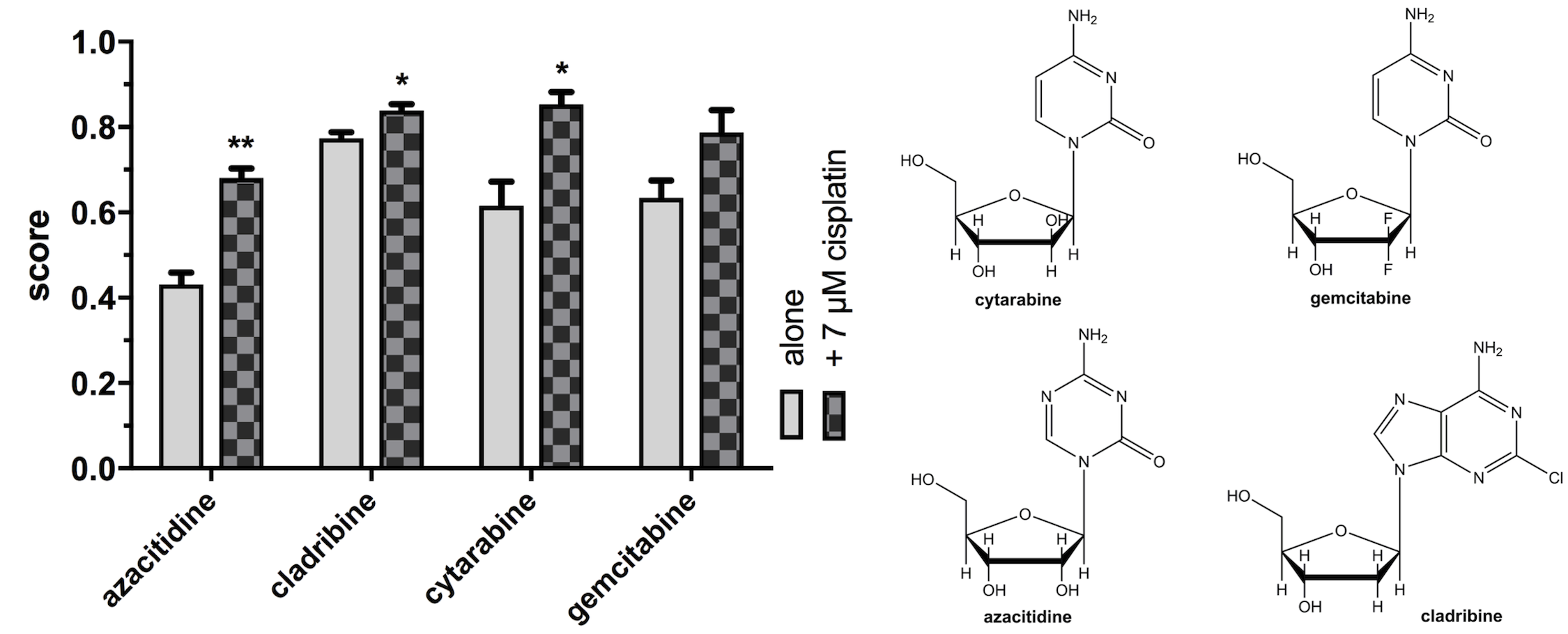

Supplement: S6 Fig — Data is presented as mean ± SD from 2 replicates (**p < 0.01 and *p < 0.05, determined by multiple (unpaired) t-tests with GraphPad Prism 7). Chemical structures (right). (TIFF) [file pone.0211268.s006.tiff]

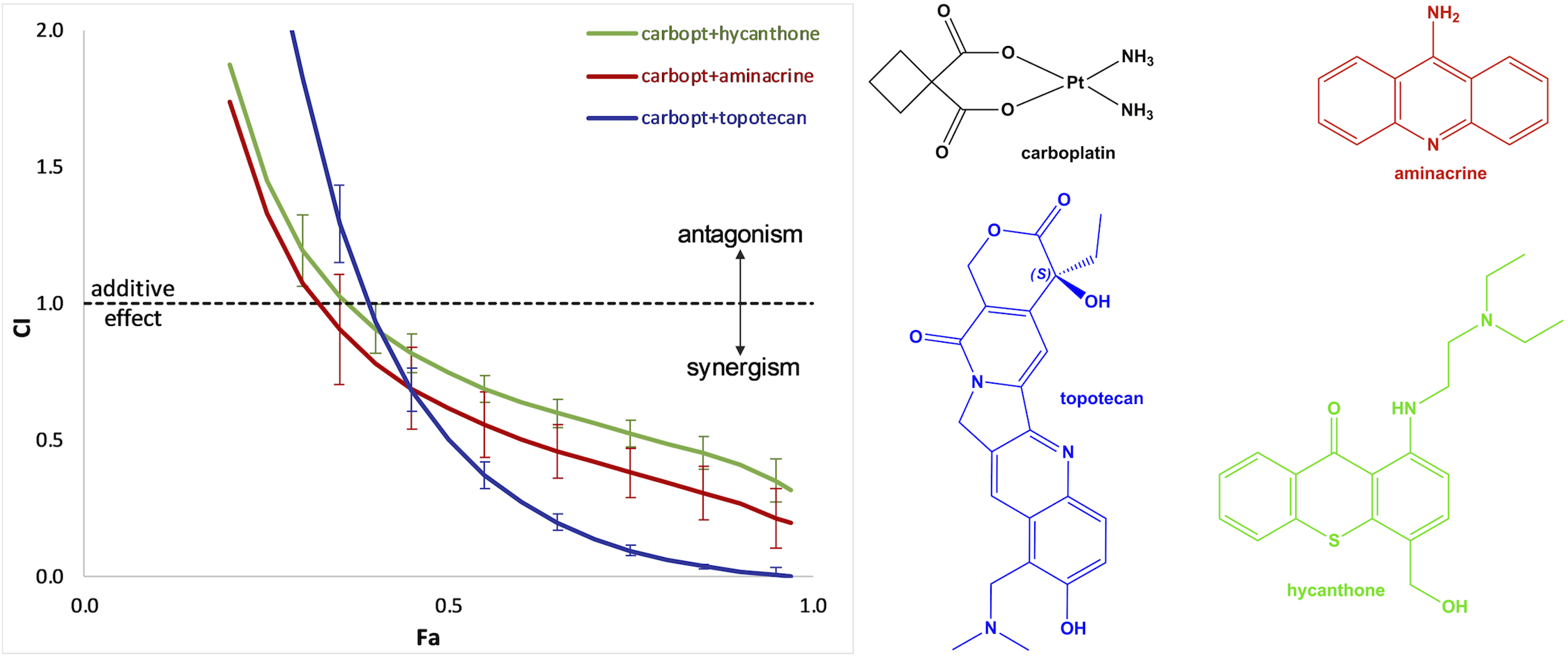

Supplement: S8 Fig — Left: Fa-CI plot of Chou-Talalay for carboplatin + topotecan (1 : 0.08; in blue), carboplatin + aminacrine (1 : 0.03; in red) and carboplatin + hycanthone (1 : 0.10; in green) combinations (72 h of exposure). Error bars represent 95% confidence intervals of the CI variability at the presented effect levels, as determined by S.D.A. Right: Chemical formulas. (TIFF) [file pone.0211268.s008.tiff]

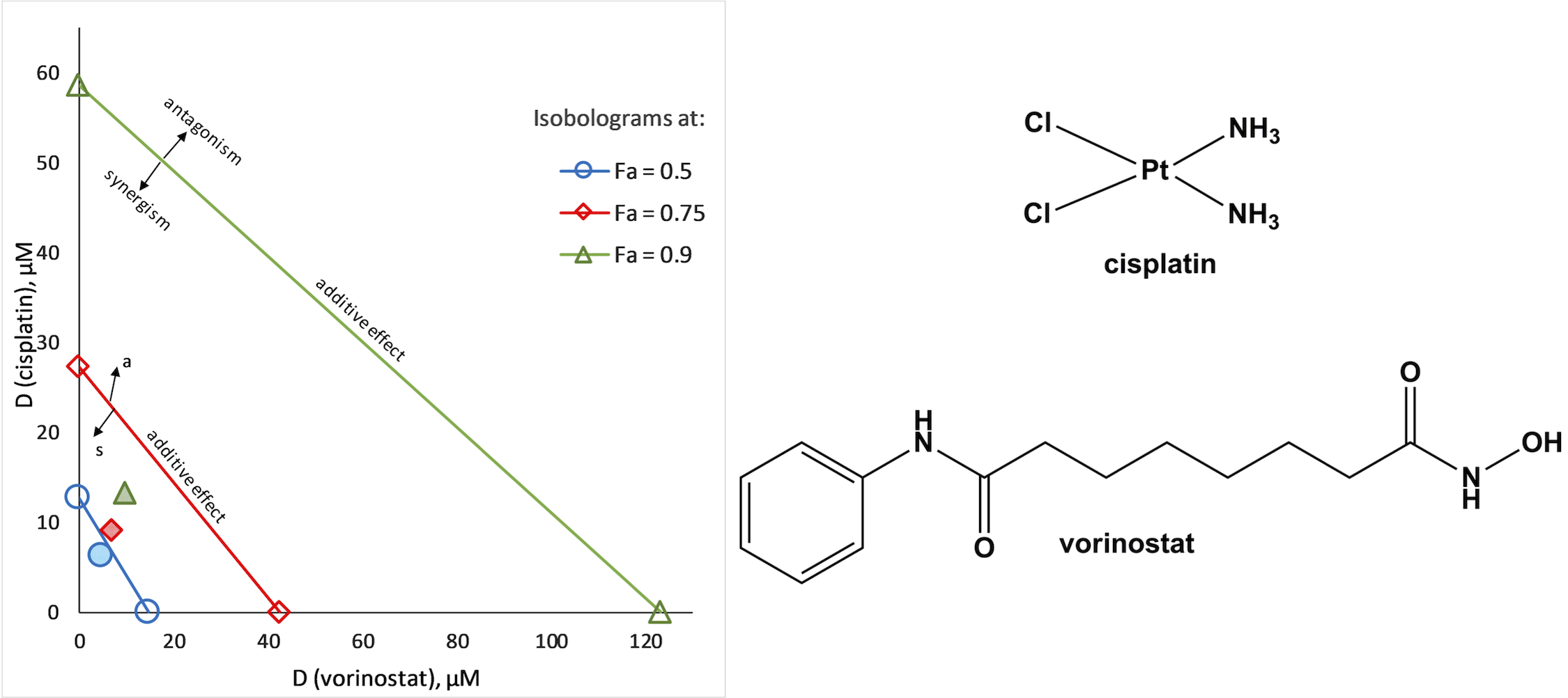

Supplement: S9 Fig — Left: classical isobologram at 0.5, 0.7 and 0.9 effect level (IC50, IC75 and IC90 concentrations, respectively). Markers for the actual combination points are pattern filled. Right: Chemical formulas. (TIFF) [file pone.0211268.s009.tiff]

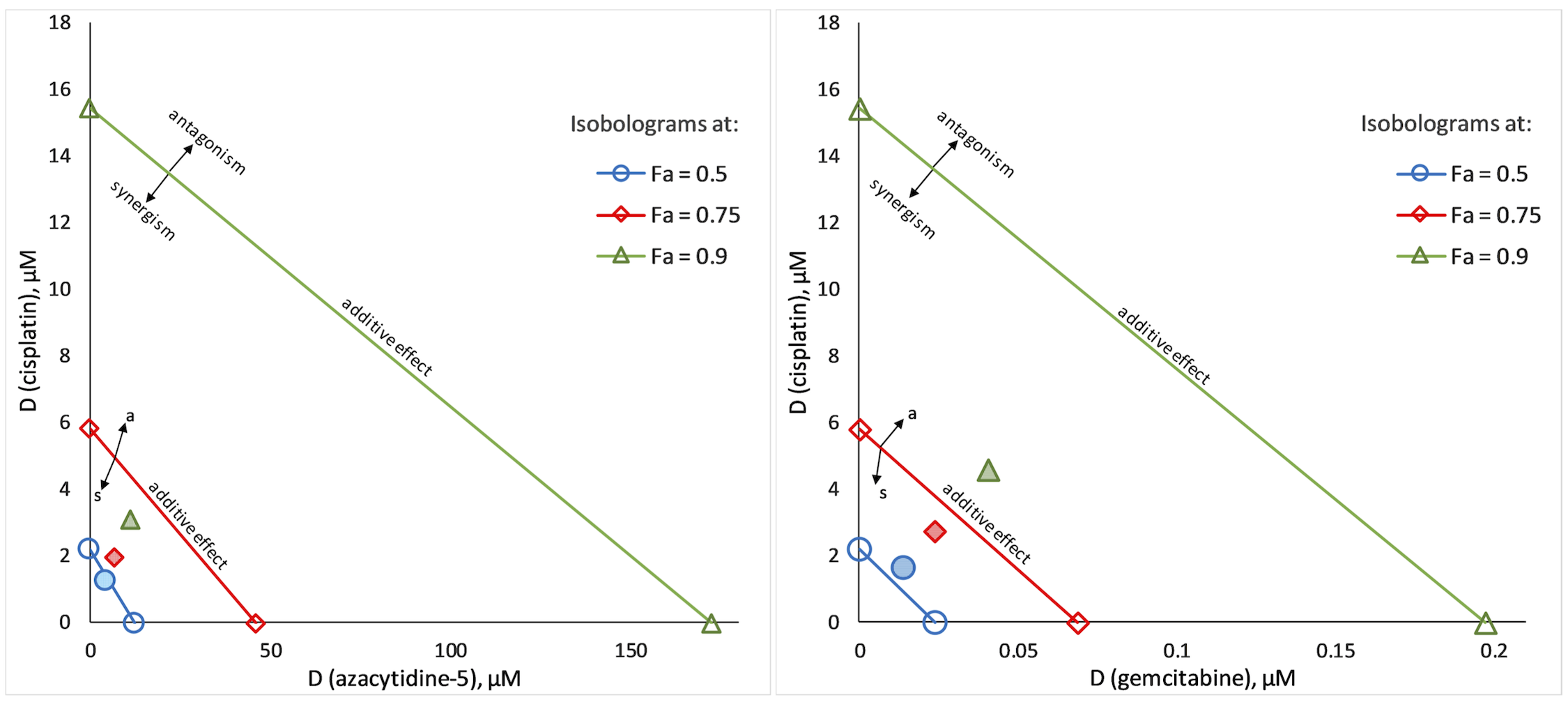

Supplement: S10 Fig — Classical isobolograms at 0.5, 0.7 and 0.9 effect level (IC50, IC75 and IC90 concentrations, respectively) for the combinations of cisplatin with azacytidine-5, 1 : 3.72 (left) and cisplatin with gemcitabine, 1 : 0.01 (right). Markers for the actual combination points are pattern filled. (TIFF) [file pone.0211268.s010.tiff]

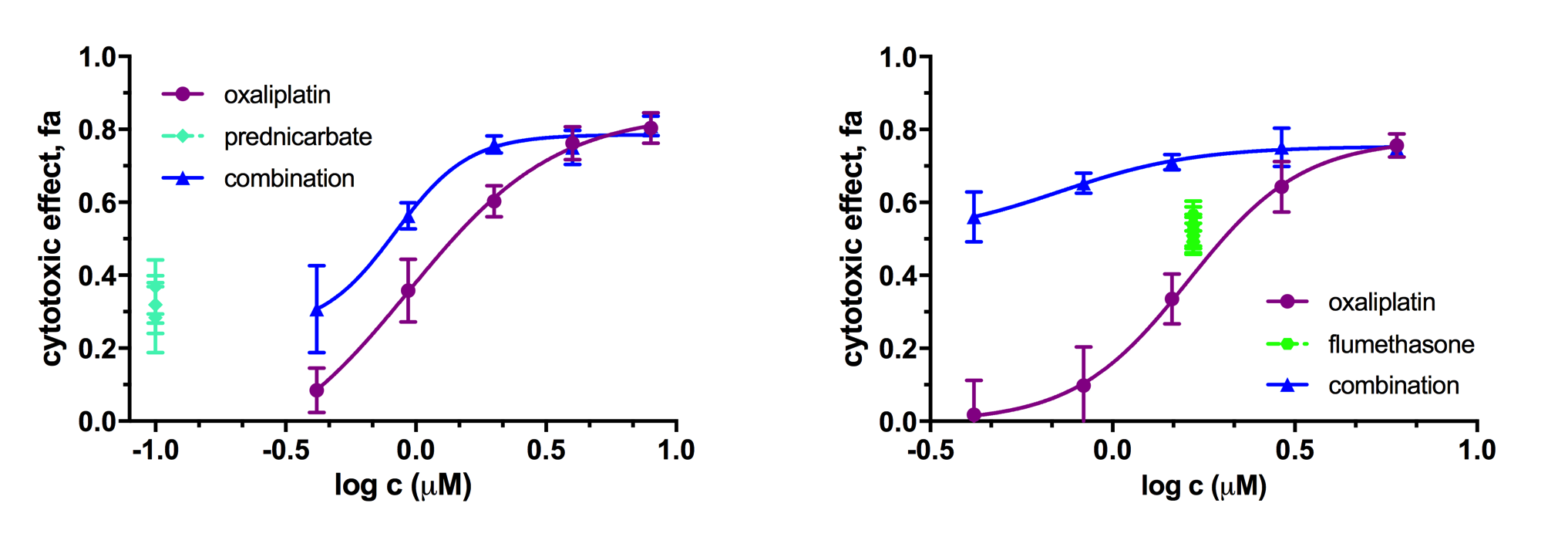

Supplement: S11 Fig — Curves fitting and graphs are prepared with GraphPad Prism 7. (TIFF) [file pone.0211268.s011.tiff]

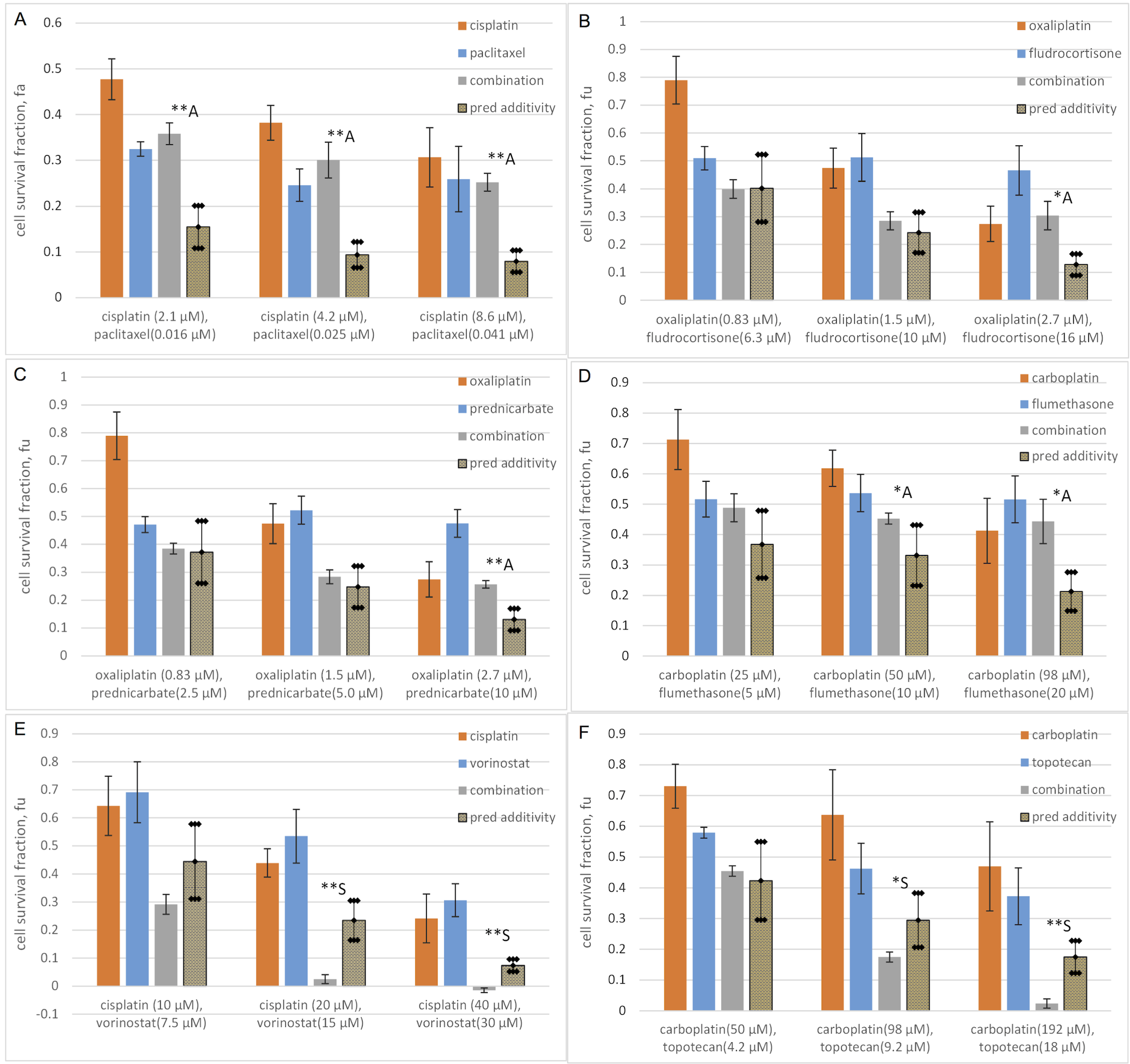

Supplement: S12 Fig — A-D): additive to antagonistic interactions between Pt drugs and corticosteroids or paclitaxel in A549 cells; E-F): synergistic interactions between cisplatin and vorinostat, and carboplatin and topotecan in PANC-1 cells. Cytotoxicity of the drugs alone and in combination at different concentrations after 72 h of continuous exposure are expressed as cell survival fractions (fu). Data is shown as mean ± SD from 4–8 replicates per concentration. Expected additive interactions for every drug combination are presented as patterned filled graph bars; error bars in this case mark the region of additivity (between 0.7 x fuA+Bcalc and 1.3 x fuA+Bcalc). A, antagonistic drug interaction (**A, denoted when: Mean (fuA+Bdet)– 1.3 x fuA+Bcalc ≥ 3 x SD (fuA+Bdet); *A, denoted when: Mean (fuA+Bdet)– 1.3 x fuA+Bcalc ≥ SD (fuA+Bdet)); S, synergistic drug interaction (**S, denoted when: 0.7 x fuA+Bcalc—Mean (fuA+Bdet) ≥ 3 x SD (fuA+Bdet); *S, denoted when: (0.7 x fuA+Bcalc—Mean (fuA+Bdet) ≥ SD (fuA+Bdet)) (TIFF) [file pone.0211268.s012.tiff]

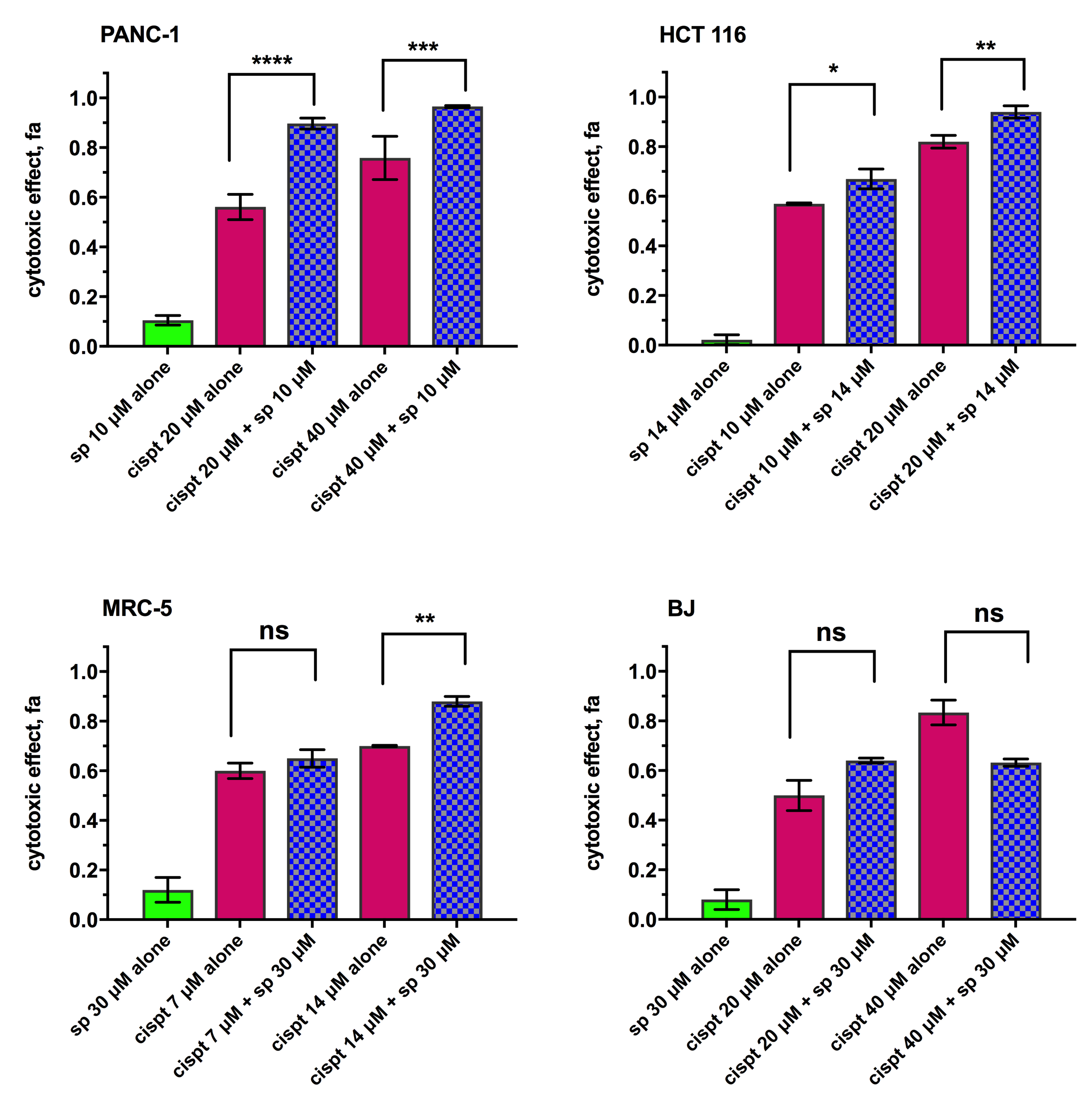

Supplement: S13 Fig — Cell viability was assessed by the PrestoBlue fluorescent assay after 72 h of continuous drugs exposure. Data is presented as mean ± SD from 2–4 replicates (combination) or 4–8 replicates (drugs alone) per concentration (****p < 0.0001, ***p < 0.001, **p < 0.01 and *p < 0.05, determined by unpaired t test with Welch’s correction). Statistical analysis, curve fitting and graphs are prepared with GraphPad Prism 7. (TIFF) [file pone.0211268.s013.tiff]

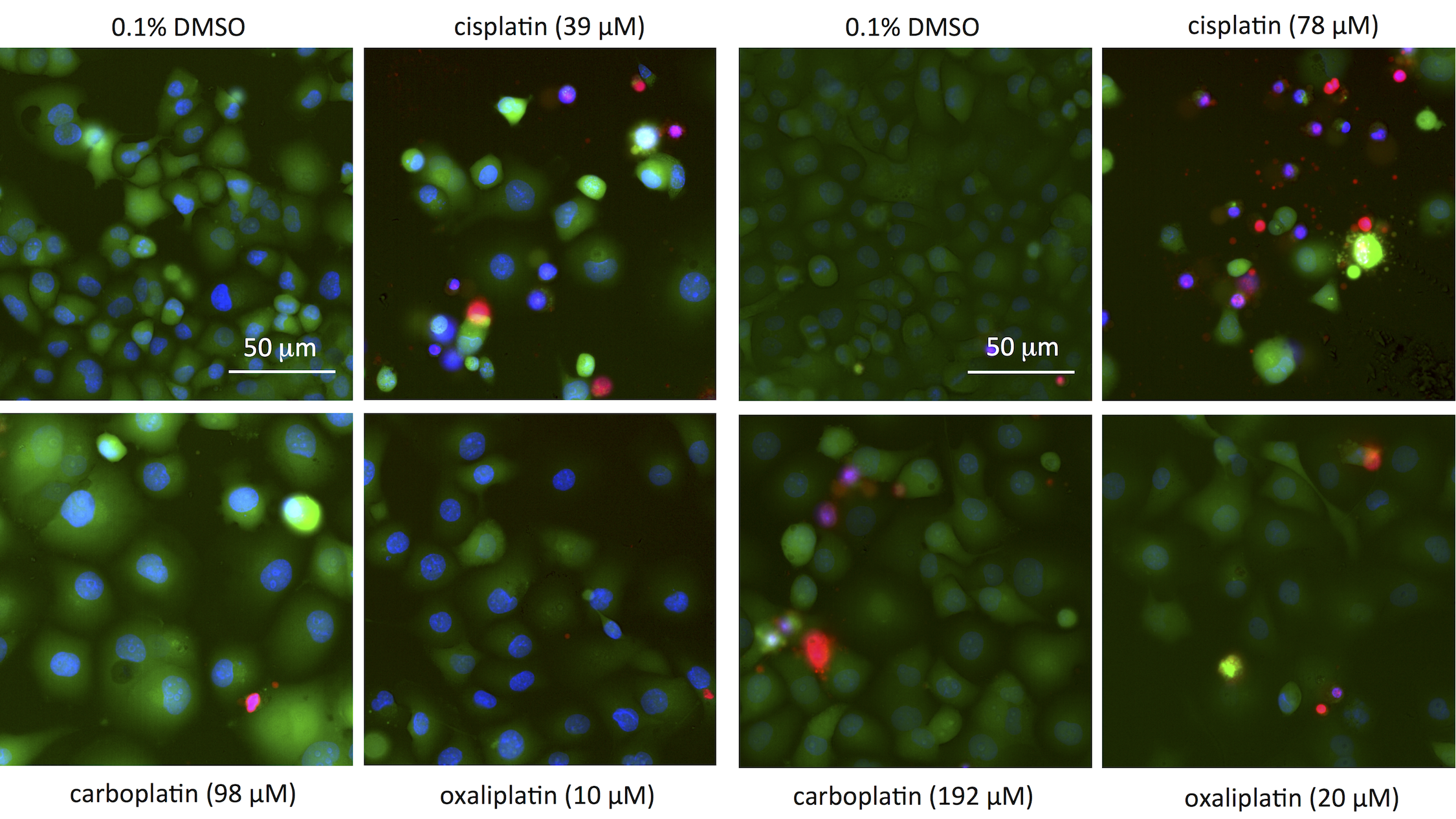

Supplement: S14 Fig — Untreated controls (cells + 0.1% DMSO) are shown for comparison. Green channel: Calcein AM (live cells), blue channel: Hoechst (nuclei), red channel: Propidium Iodide (dead cells). (TIFF) [file pone.0211268.s014.tiff]

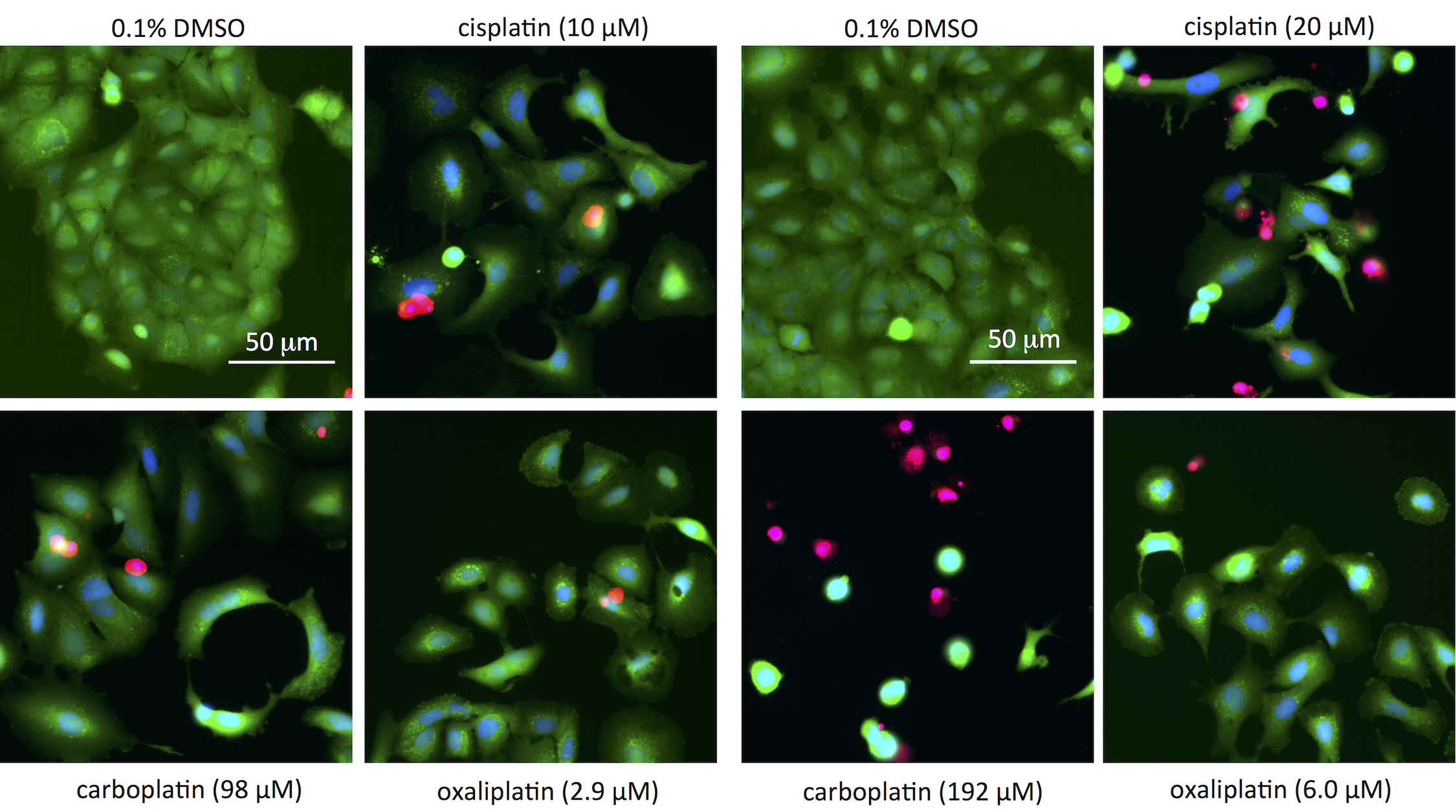

Supplement: S15 Fig — Untreated controls (cells + 0.1% DMSO) are shown for comparison. Green channel: Calcein AM (live cells), blue channel: Hoechst (nuclei), red channel: Propidium Iodide (dead cells). (TIFF) [file pone.0211268.s015.tiff]

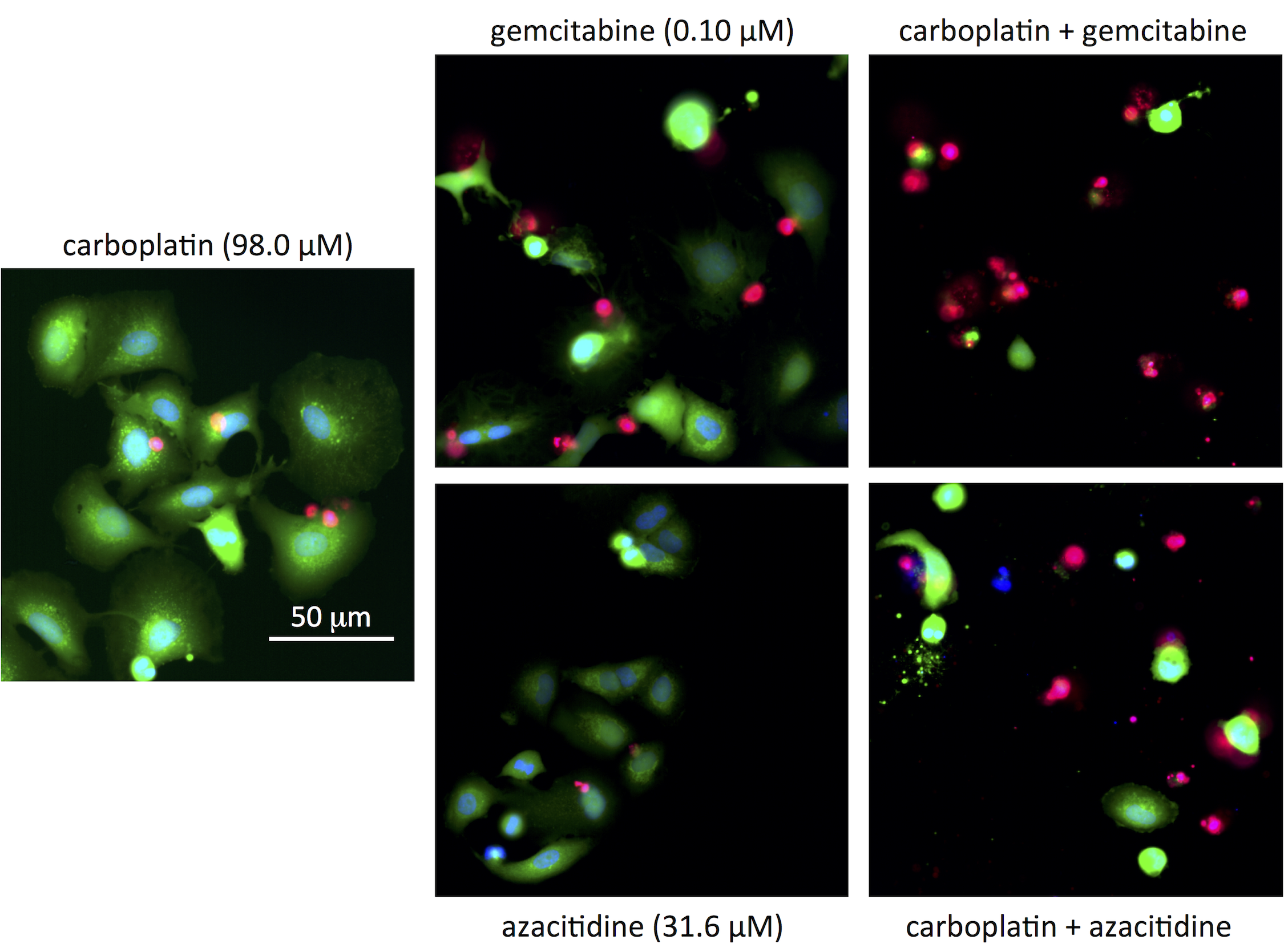

Supplement: S16 Fig — Green channel: Calcein AM (live cells), blue channel: Hoechst (nuclei), red channel: PI (dead cells). (TIFF) [file pone.0211268.s016.tiff]

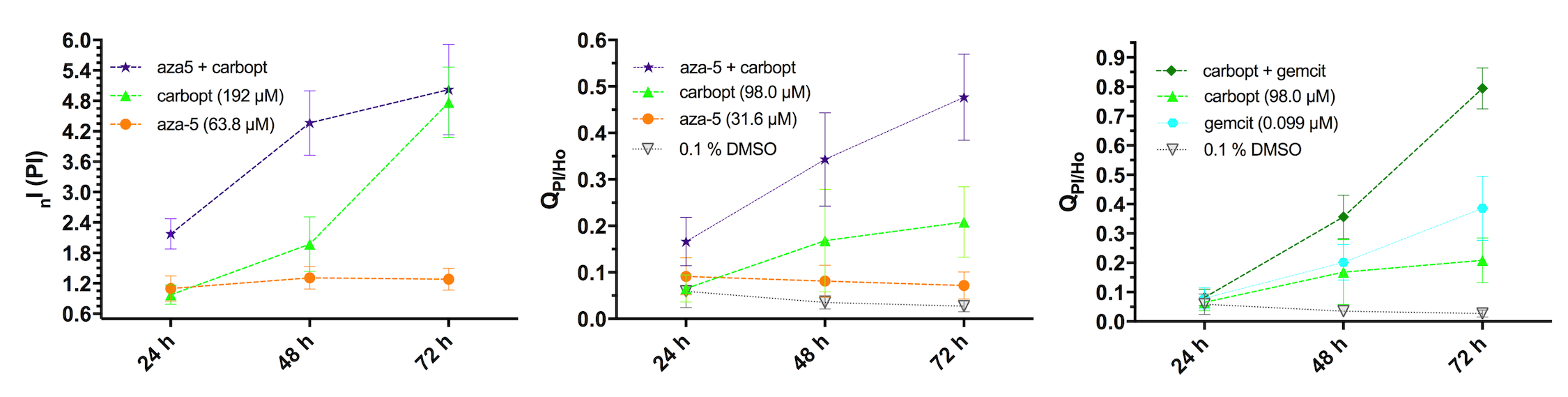

Supplement: S17 Fig — Synergistic combinations between carboplatin and azacytidine-5 (left, center) or gemcitabine (right) in A549 cells. Time dependent increase of nI(PI) (left) and QPI/Ho (center, right) after treatment with the drugs alone and in combinations. Values are obtained as mean ± SD from at least 2 wells/condition and 9 field of view/well. (TIFF) [file pone.0211268.s017.tiff]

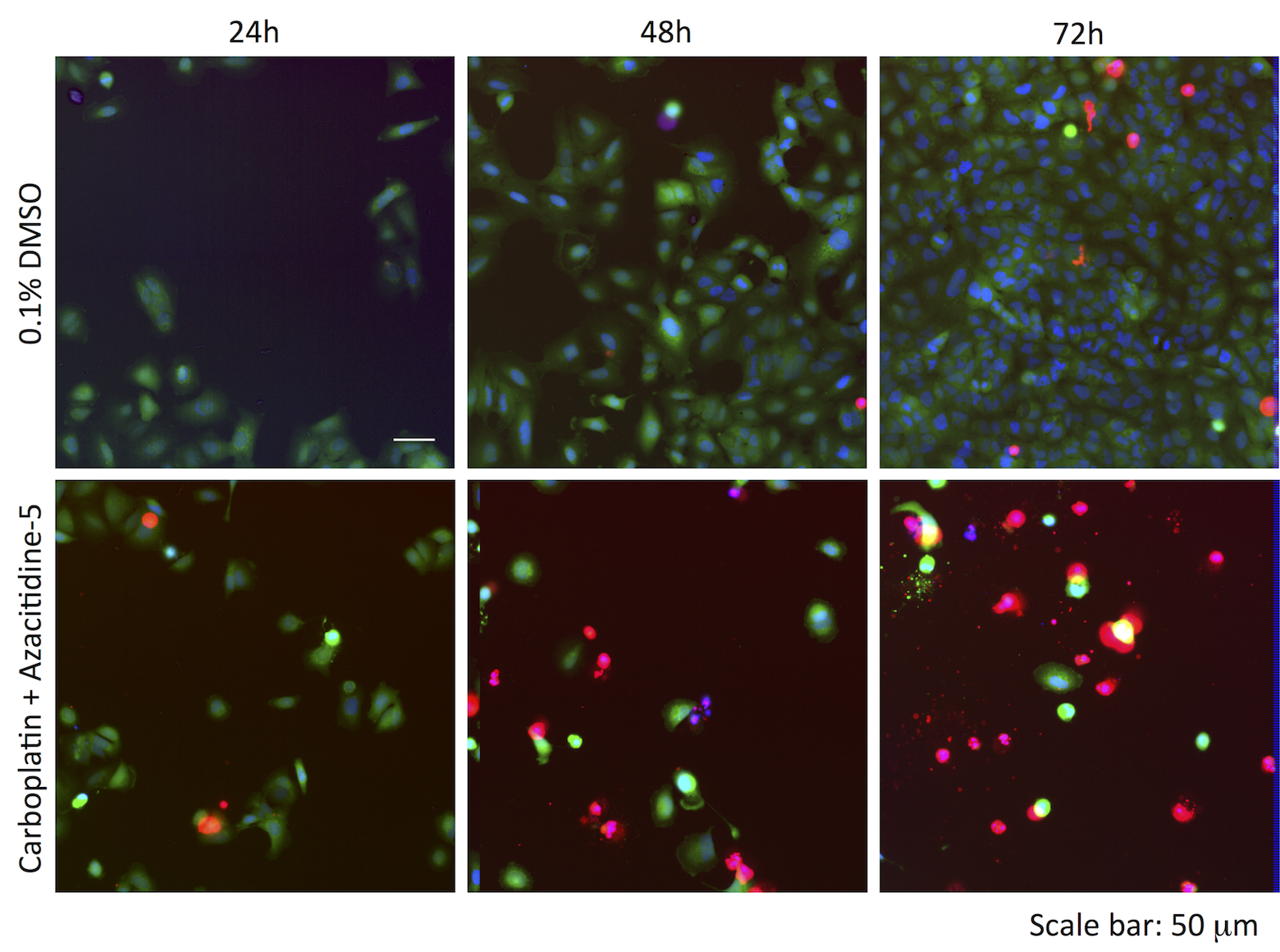

Supplement: S18 Fig — Untreated controls (cells + 0.1% DMSO) are shown for comparison. Green channel: Calcein AM (live cells), blue channel: Hoechst (nuclei), red channel: PI (dead cells). (TIFF) [file pone.0211268.s018.tiff]

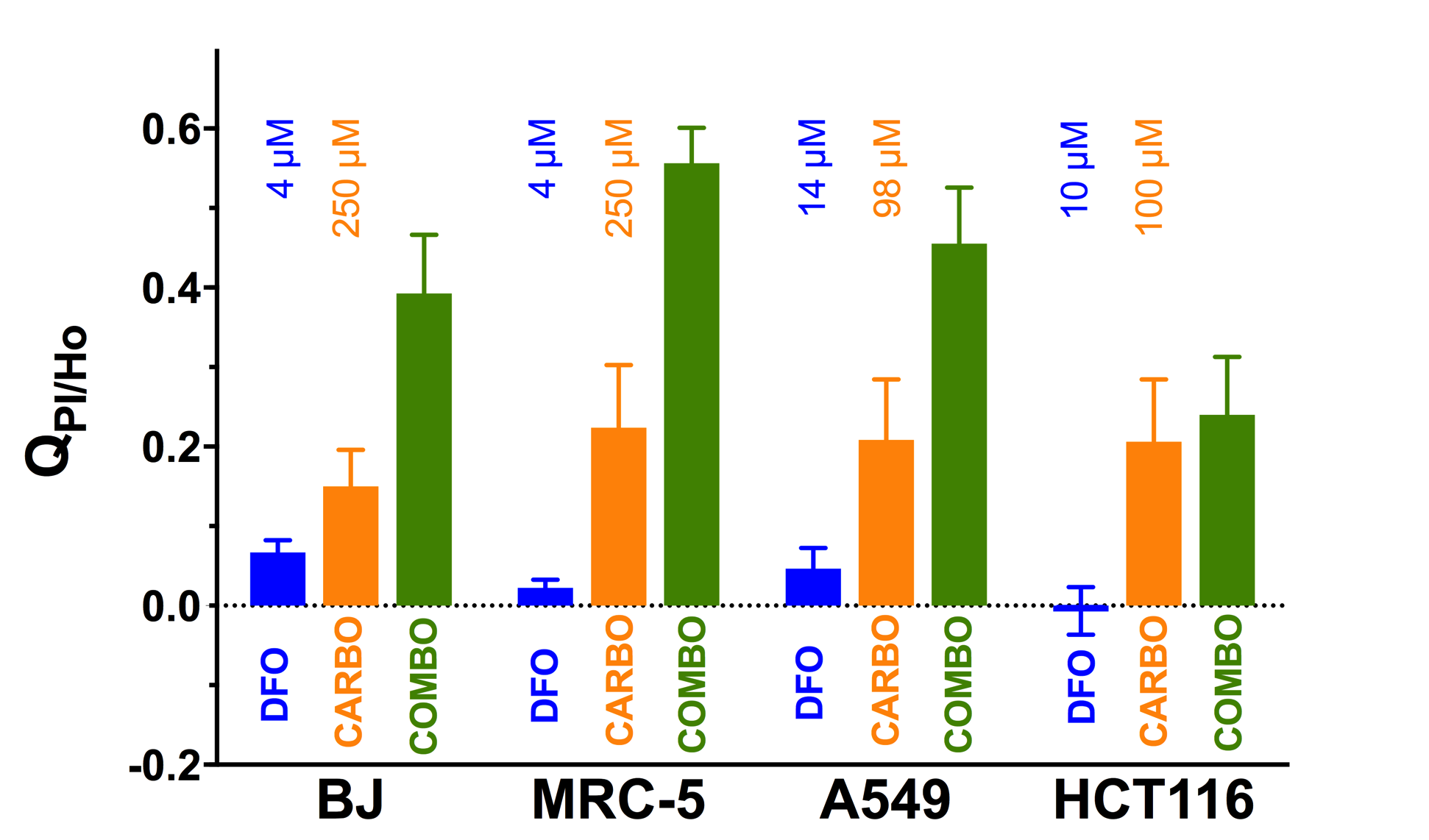

Supplement: S19 Fig — Values are acquired as mean ± SD from at least 2 wells/condition and 9 field of view/well. Carboplatin concentrations at which the drug has similar potency in the different cell lines were chosen for the experiment in order to facilitate comparisons. (TIFF) [file pone.0211268.s019.tiff]

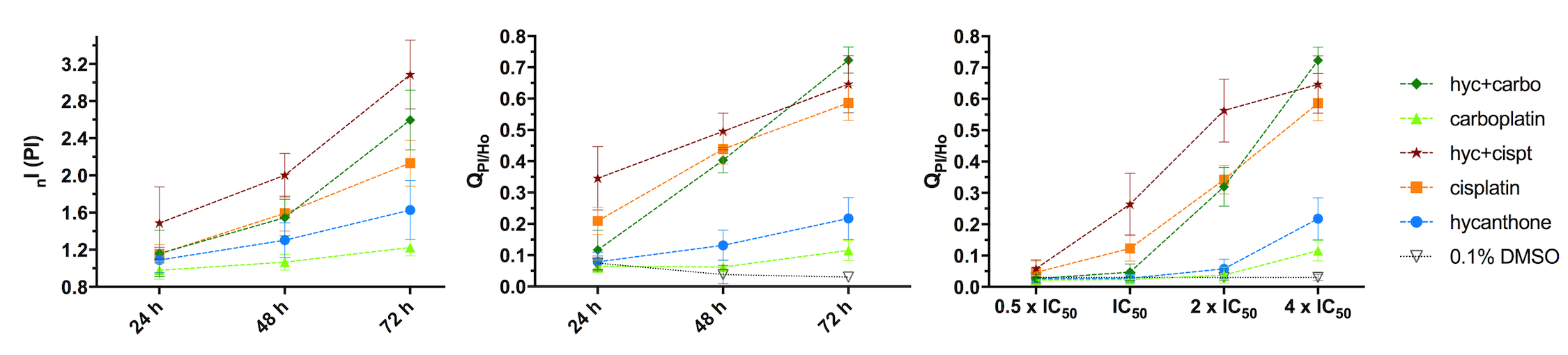

Supplement: S20 Fig — Time dependent increase of nI(PI) (left) and QPI/Ho (center) after treatment with the drugs alone (hycanthone, 15.8 μM; cisplatin, 77.5 μM and carboplatin, 192 μM) and in combinations. Right: dose-dependent increase of QPI/Ho after 72 h exposure to the drugs alone (in concentrations ranging from ½ x IC50 to 4 x IC50) and their combinations at a fixed concentration ratio. Values are obtained as mean ± SD from at least 2 wells/condition and 9 field of view/well. (TIFF) [file pone.0211268.s020.tiff]

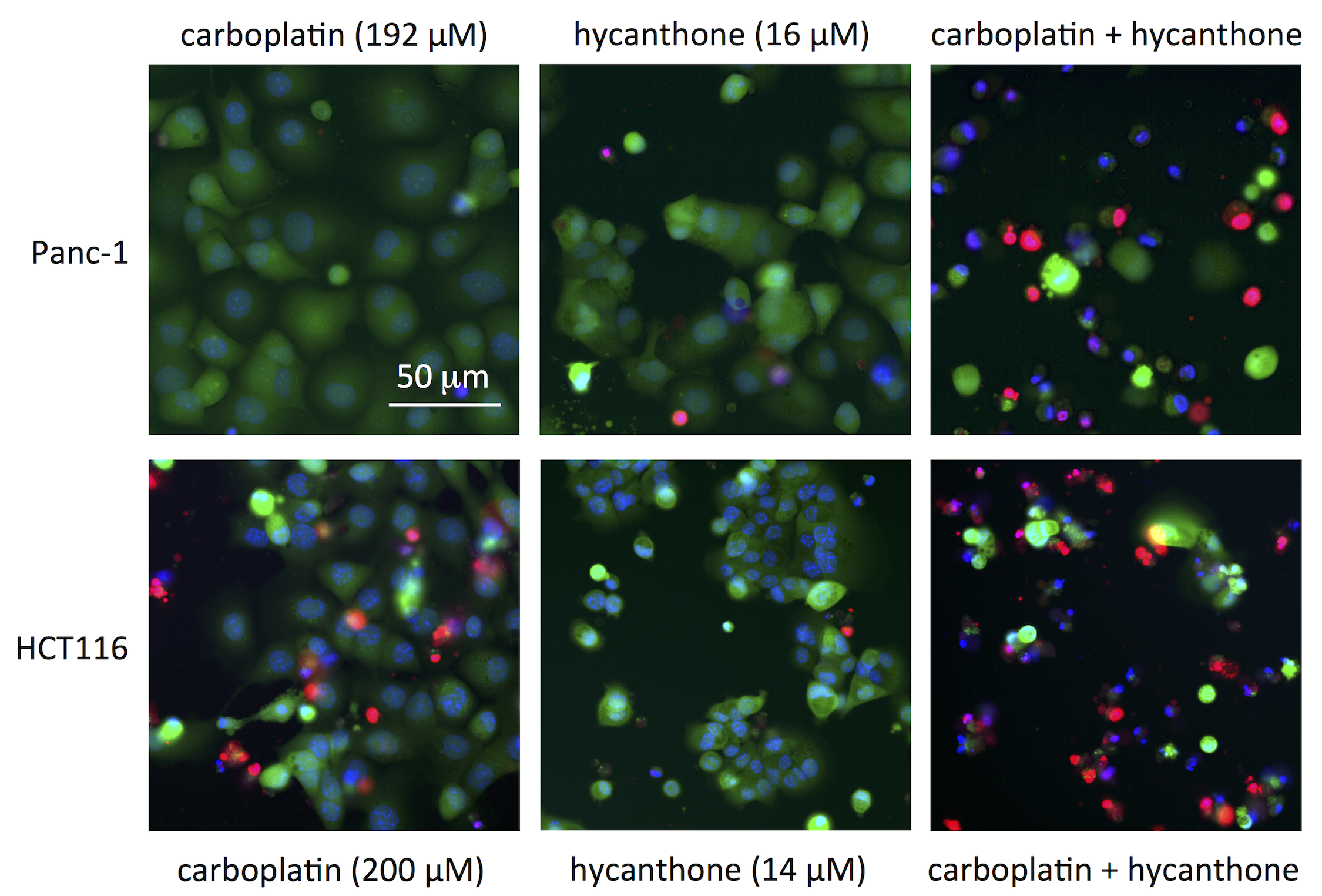

Supplement: S21 Fig — Images of PANC-1 (top) and HCT116 (bottom) cells after 72 h of exposure to hycanthone, carboplatin and their combination. Green channel: Calcein AM (live cells), blue channel: Hoechst (nuclei), red channel: Propidium Iodide (dead cells). (TIFF) [file pone.0211268.s021.tiff]

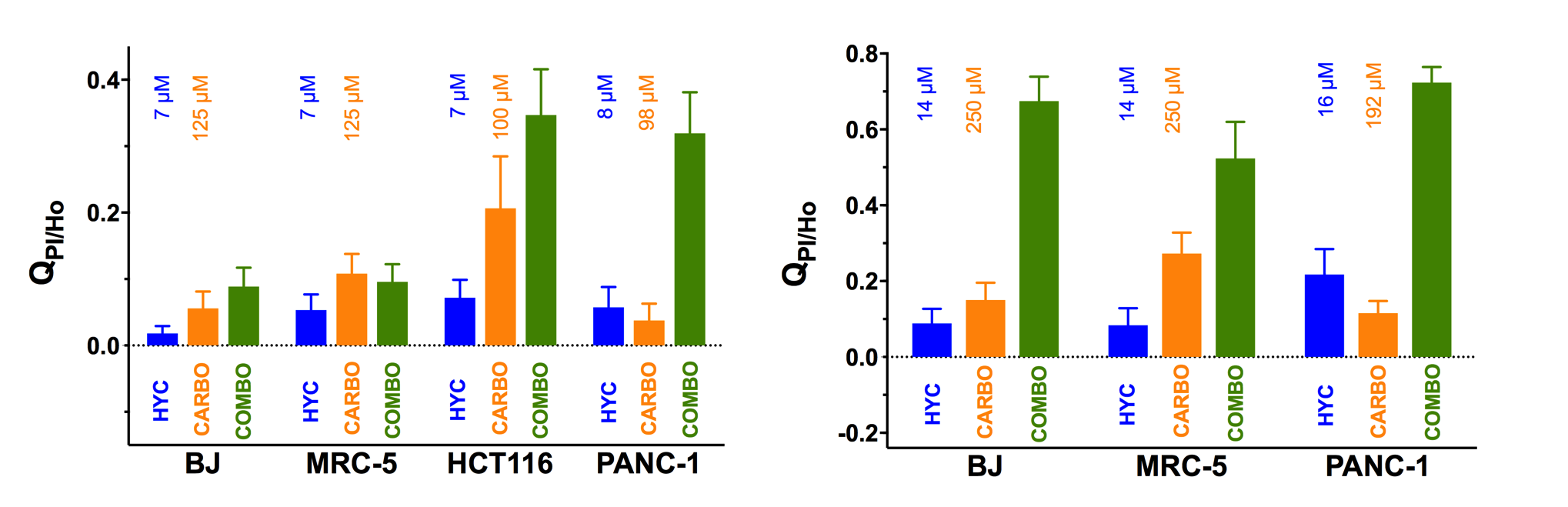

Supplement: S22 Fig — Comparison of QPI/Ho obtained for PANC-1, MRC-5, BJ and HCT116 cells exposed to carboplatin, hycanthone and their combination for 72 h. Values are acquired as mean ± SD from at least 2 wells/condition and 9 field of view/well. (TIFF) [file pone.0211268.s022.tiff]

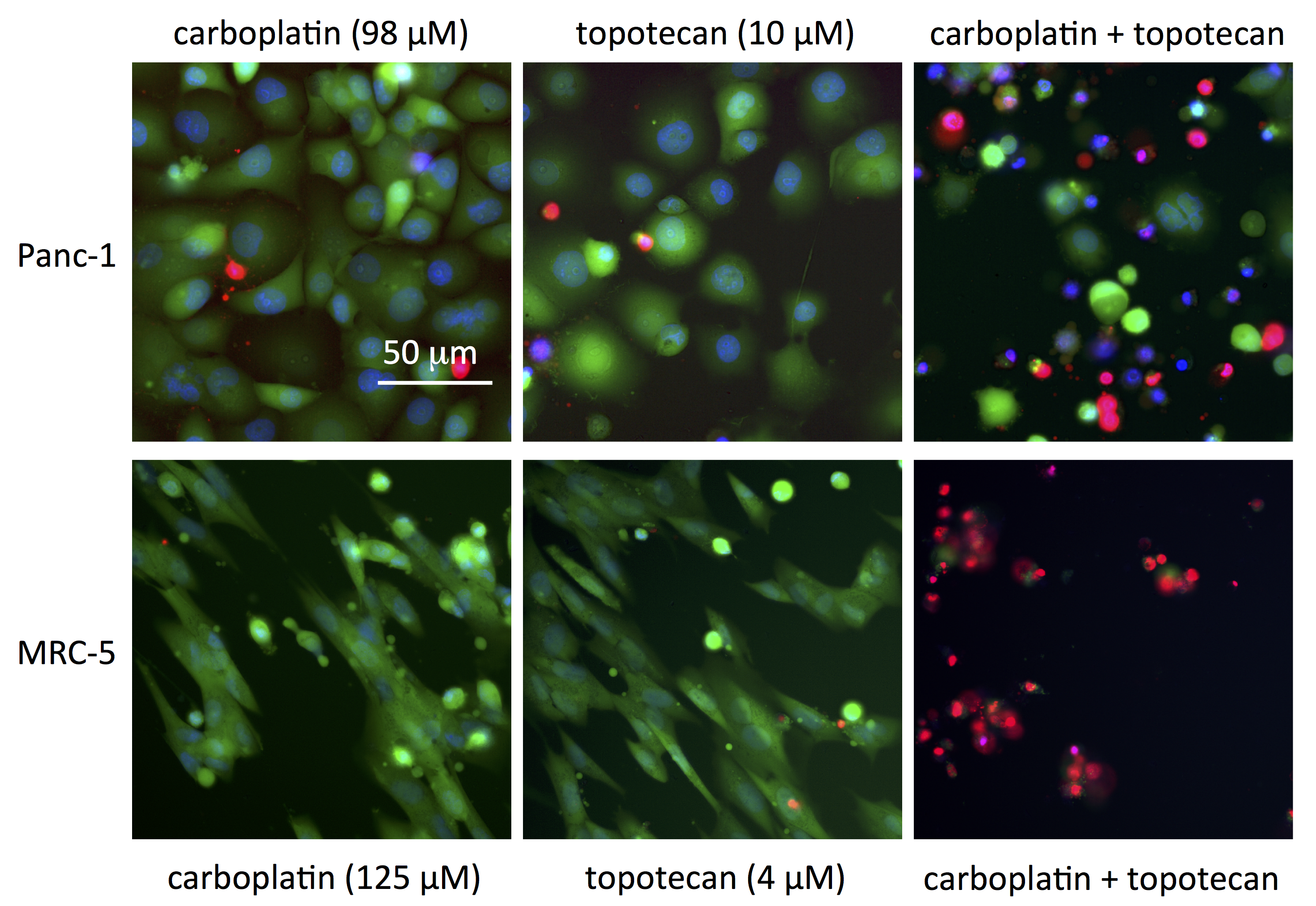

Supplement: S23 Fig — Images of PANC-1 (top) and MRC-5 cells (bottom) after 72 h of exposure to carboplatin, topotecan and their combination. Green channel: Calcein AM (live cells), blue channel: Hoechst (nuclei), red channel: Propidium Iodide (dead cells). (TIFF) [file pone.0211268.s023.tiff]

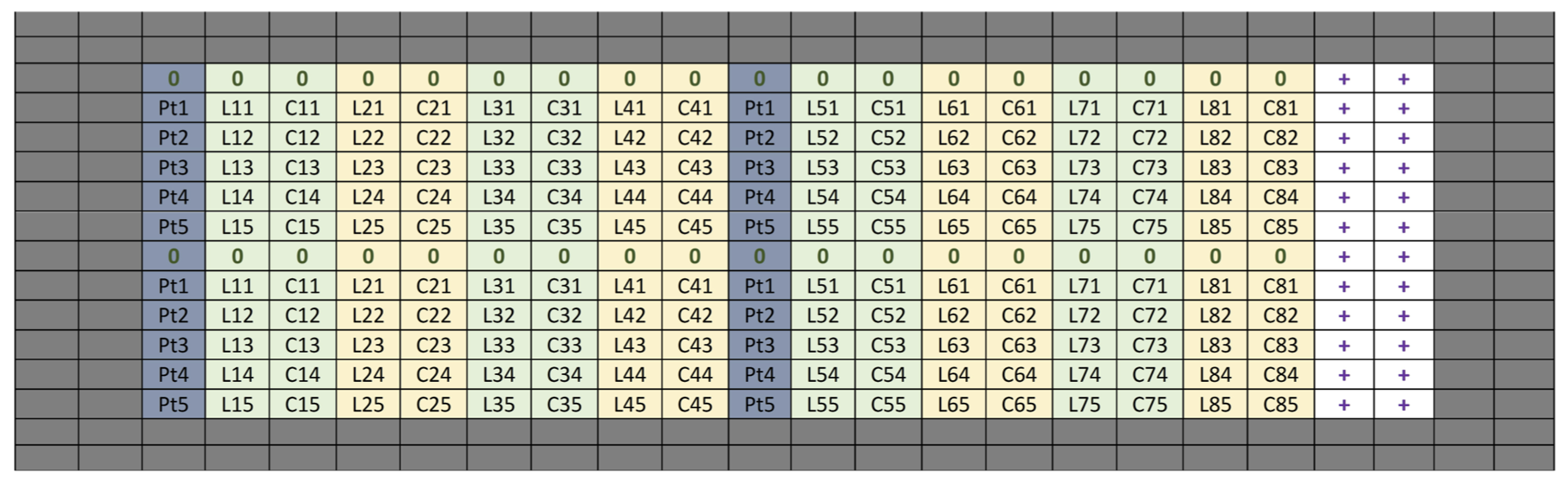

Supplement: S24 Fig — First and last two rows and columns are drug-free (cells only), columns with ‘+’ are the positive controls (20 μM Doxorubicin.HCl, 0.2% DMSO), rows ‘0’ are the negative controls (0.2% DMSO); Pt(1–5): the respective Pt drug in five concentrations, Lx(1–5): library compound in five concentrations, Cx(1–5): combination of the Pt and the PCL compound at the same concentrations (i.e. ¼ IC50, ½ IC50, IC50, 2 IC50 and 4 IC50 of the respective compounds). DMSO is added to all wells to reach the concentration of the control wells. Total volume per well after seeding the cells is 30 μl. Eight drug combinations can be tested on one such plate. Every plate is prepared in duplicate. (TIFF) [file pone.0211268.s024.tiff]
