# Supplementary material for: Screening-based approach to discover effective platinum-based chemotherapies for cancers with poor prognosis
Source: PLoS One. 2019 Jan 29;14(1):e0211268. doi: 10.1371/journal.pone.0211268 (PMC6350982; doi:10.1371/journal.pone.0211268)
Supplement: S2 Fig — (PDF) [file pone.0211268.s002.pdf]

DMSO and Pt(II) complexes

Significant decrease of the activity of cisplatin in both PANC-1 and A549 cells was detected when cisplatin (concentrations between 2 and 80 μM, corresponding to volumes of 30 to 240 nl, in PBS) and DMSO (20 to 60 nl) were dispensed (Echo 550 acoustic liquid handler) concurrently in the empty 384-well plates before cell seeding. When cisplatin was added manually, after cells seeding in medium (final DMSO concentration < 0.2%), no effect on its cytotoxicity was observed (S2 Fig, A).

Oxaliplatin and carboplatin (at much slower rate) react with DMSO in aqueous media[1]; nevertheless, the effects of DMSO on the cytotoxicity of carboplatin and oxaliplatin in A549 and PANC-1 cells were only minor (S2 Fig, B and C).

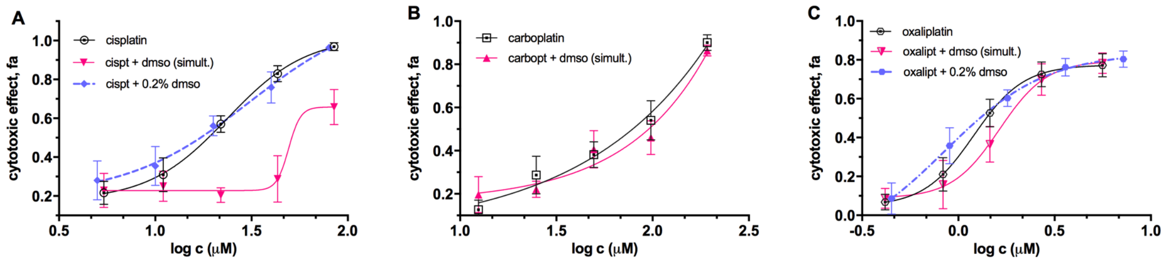

S2 Fig. Effect of DMSO on the cytotoxicity of cisplatin (A) in PANC-1 cells, carboplatin (B) and oxaliplatin (C) in A549 cells after 72 h of incubation (as determined by the PrestoBlue fluorescent assay). Pt drugs were dispensed either concurrently with DMSO (30 nl, final concentration of DMSO, 0.1 %) before cell seeding or last, after cell seeding (final concentration of DMSO, 0.2 %).

[1] Varbanov H, Ortiz D, Höfer D, Menin L, Galanski M, Keppler B, Dyson P (2017) Oxaliplatin reacts with DMSO only in the presence of water. Dalton Trans. 46: 8929-8932
